# Differential Modulation of Keratinocyte Proliferation, Differentiation, and Barrier Function by Hemp Oil, Blackcurrant Seed Oil, and Vitamin D_3_ Under Atopic‐Associated Cytokine Stimulation

**DOI:** 10.1002/biof.70137

**Published:** 2026-08-10

**Authors:** Daniel Tortolani, Lucia Scipioni, Alessandro Anselmucci, Francesca Ciaramellano, Meri Di Leonardo, Isa Fusaro, Sergio Oddi, Alessandro Gramenzi

**Affiliations:** ^1^ Department of Veterinary Medicine University of Teramo Teramo Italy; ^2^ European Center of Brain Research/Fondazione Santa Lucia IRCCS Rome Italy; ^3^ Azienda Sanitaria Locale di Teramo Teramo Italy

**Keywords:** atopic dermatitis, blackcurrant seed oil, canine keratinocytes, differentiation, epithelial barrier, hemp oil, nutraceutical adjuvant, oxidative stress, proliferation, vitamin D3

## Abstract

Atopic dermatitis (AD) is a chronic inflammatory skin disease characterized by keratinocyte hyperproliferation, altered differentiation, barrier dysfunction, and oxidative stress. Currently available therapies, including monoclonal antibodies and JAK inhibitors, have improved AD management but do not address all aspects of the disease, supporting the investigation of natural‐product strategies as complementary approaches to long‐term care. An acute in vitro model of canine atopic‐like inflammation was established by exposing canine progenitor epidermal keratinocytes (CPEK) for 24 h to a defined cytokine cocktail (IFN‐γ, IL‐4, IL‐13), followed by 24 h of treatment with hemp oil, blackcurrant seed oil, vitamin D_3_, or their combination. Proliferation (Ki‐67, cell‐cycle), differentiation (KRT5, KRT10, TGM1, involucrin), tight‐junction organization (CLDN1, TJP1, ZO‐1, TEER), wound closure, nitrosative stress (3‐nitrotyrosine), the NRF2/BACH1/HMOX1 axis, and the secretion of STAT1‐ and NF‐κB‐dependent inflammatory mediators (CXCL9, CXCL10, IL‐8, IL‐6) and the STAT6‐targeted chemokine CCL17 were assessed. The cytokine cocktail induced a coherent AD‐like phenotype: increased Ki‐67, downregulated CLDN1, sustained nitrosative stress, NRF2 elevation paralleled by BACH1 induction, and robust secretion of STAT1‐ and NF‐κB‐dependent mediators. The four treatments modulated this phenotype according to clearly differential, pathway‐specific profiles. Vitamin D_3_, alone or combined, emerged as the most effective modulator of proliferation and acted preferentially on the downstream redox arm, inducing HMOX1 and reducing BACH1; both vitamin D_3_‐containing formulations exerted broad anti‐inflammatory activity across the STAT1 and NF‐κB axes. Hemp oil acted preferentially on tight‐junction integrity (CLDN1 recovery), on the upstream NRF2 arm (NFE2L2 induction), on the STAT1 chemokine arm (CXCL9, CXCL10 reduction), and on wound closure. Blackcurrant seed oil acted preferentially on NF‐κB‐dependent IL‐8 and on early wound‐closure dynamics. This differential pharmacological footprint provides a rational basis for further investigation of these formulations as nutraceutical adjuncts in canine AD.

## Graphical abstract

In cytokine‐stimulated canine keratinocytes, hemp oil strengthened tight junctions and migration, blackcurrant seed oil normalized differentiation, and vitamin D_3_ curbed proliferation, each also shaping distinct inflammatory pathways. Combined, they acted complementarily across multiple levels, supporting a rational adjuvant strategy for canine atopic dermatitis.

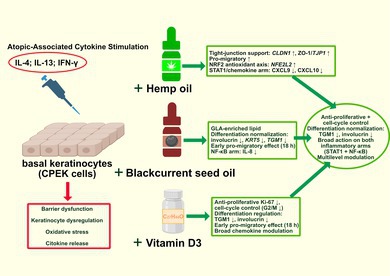

## 1 Introduction

Atopic dermatitis (AD) is a multifactorial, chronic inflammatory skin disease affecting both humans (hAD) and dogs (cAD), characterized by dysregulated immune responses, keratinocyte hyperproliferation, abnormal differentiation, barrier dysfunction, and oxidative stress [1, 2, 3, 4, 5]. A central feature of cAD pathogenesis is the disruption of epidermal barrier integrity. Like hAD, cAD presents reduced expression of superficial adhesion molecules within the stratum corneum, together with alterations involving the basal layer, where tight junction proteins such as claudin‐1 and zonula occludens‐1 (ZO‐1) are critically impaired [6, 7, 8]. In parallel, changes in epidermal lipid composition [9] and abnormal keratinocyte differentiation [10] exacerbate transepidermal water loss (TEWL), facilitate allergen and microbial penetration, and perpetuate chronic inflammatory responses [11].

These structural alterations are mostly driven by the inflammatory environment acting directly on the keratinocyte, which is now recognized not as a passive target but as an active participant in AD pathology. Th2‐derived cytokines, particularly IL‐4 and IL‐13, signal in keratinocytes mainly through the type II receptor complex (IL‐4Rα/IL‐13Rα1), engaging Janus kinase 1 (JAK1), TYK2, and JAK2 and the downstream transcription factors STAT6 and STAT3 [12]. Activation of this JAK–STAT6/STAT3 axis suppresses the keratinocyte terminal differentiation program, downregulating filaggrin, loricrin, involucrin, and keratins 1 and 10 in mouse and human [13, 14, 15]. In addition, IL‐4 and IL‐13 have been reported to reduce the expression of tight‐junction components, including claudin‐1, further weakening epidermal cohesion [13, 14, 15]. This cytokine‐driven inflammation is not confined to the Th2 arm: the Th1 cytokine IFN‐γ, also detected in atopic skin, activates the JAK1/STAT1 pathway in keratinocytes and cooperates with Th2 signals to sustain a mixed Th1/Th2 inflammatory environment, while the pruritogenic cytokine IL‐31, a key mediator of both hAD and cAD, further perturbs keratinocyte differentiation and barrier homeostasis [4, 16].

In parallel, oxidative stress plays a central role: reactive oxygen species (ROS) and downstream stress signaling worsen keratinocyte damage, further compromising barrier repair and promoting inflammation [17, 18, 19]. The cellular response to this redox imbalance is largely orchestrated by the KEAP1–NRF2 system, a thiol‐based sensor–effector apparatus that, upon oxidative challenge, drives the transcription of cytoprotective and antioxidant genes such as heme oxygenase‐1 (HMOX1) [20, 21]. However, in atopic skin this defensive program is frequently unable to fully counteract the sustained oxidative burden, and the activity of NRF2‐dependent responses in the AD epidermis has been reported to be functionally compromised [20, 21]. This relative insufficiency of endogenous antioxidant defenses provides a rationale for adjunctive interventions aimed at reinforcing the NRF2 axis.

Current therapies for cAD include topical corticosteroids, immunomodulators, and, more recently, monoclonal antibodies targeting specific cytokines or Janus kinase (JAK) pathway inhibitors [5, 22, 23, 24]. These biologics are effective but expensive, may have side effects, and do not always fully restore normal barrier function or keratinocyte homeostasis. There is therefore a pressing need for innovative topical approaches based on natural compounds, which are already recognized as safe and represent commercially affordable alternatives to complement current therapies by targeting barrier protection.

There is therefore a pressing need for innovative topical approaches based on natural compounds, already recognized as safe and representing commercially affordable alternatives, to complement current therapies. In this context, vitamin D_3_, cannabidiol (CBD)‐rich hemp oil, and γ‐linolenic acid (GLA)‐rich blackcurrant seed oil (BCS oil) are of particular interest, as each of them has been reported to act through distinct mechanisms on the inflammatory, redox, and barrier‐related processes that, as outlined above, are central to cAD pathogenesis.

Vitamin D_3_ (cholecalciferol) is one such promising adjuvant. In a placebo‐controlled, double‐blinded, randomized trial in dogs with AD, systemic administration of vitamin D_3_ significantly reduced pruritus and lesion severity, and these clinical improvements correlated with increased serum 25‐hydroxyvitamin D levels [25]. Beyond its well‐established immunomodulatory actions, including the enhancement of antimicrobial peptides, which are often deficient in AD [26], vitamin D_3_ also exerts relevant antioxidant and barrier‐supporting effects in the skin. Acting through the vitamin D receptor (VDR), it has been shown to activate the NRF2 pathway and to induce the expression of antioxidant enzymes, including heme oxygenase‐1, while reducing ROS generation in keratinocytes [27]. In parallel, vitamin D_3_ reinforces epithelial cohesion by promoting the expression of tight‐junction proteins such as ZO‐1 and occludin, with a measurable increase in transepithelial electrical resistance [28].

Cannabidiol (CBD), the non‐psychoactive phytocannabinoid derived from *Cannabis sativa* , and the major bioactive constituents of hemp oil has recently garnered significant interest due to its anti‐inflammatory, antipruritic, and antioxidant properties [29]. Beyond the canonical CB_1_/CB_2_ axis of the cutaneous endocannabinoid system (ECS), CBD engages a broader receptor landscape (e.g., TRP channels, PPARs, adenosine receptors) and can modulate downstream inflammatory signaling (e.g., NF‐κB), pruritus pathways, and redox balance processes central to AD pathophysiology [29, 30]. Of relevance, CBD has been shown to act directly on the antioxidant machinery of keratinocytes: in primary human keratinocytes it induces several NRF2 target genes, with heme oxygenase‐1 (HMOX1) being the most strongly upregulated, through nuclear export and proteasomal degradation of the transcriptional repressor BACH1 [31]. Preliminary veterinary data suggest clinically relevant benefits on itch and lesional severity with topical or oral cannabinoid‐based interventions, while safety evaluations generally report good tolerability for CBD in dogs, with mostly mild and self‐limited adverse events [32, 33].

On the other hand, γ‐linolenic acid (GLA), primarily supplied by BCS oil, has been investigated for its potential benefits in atopic dermatitis. As an omega‐6 fatty acid, GLA bypasses the rate‐limiting Δ6‐desaturase step, an enzymatic activity reported to be deficient in the epidermis, and is readily elongated to dihomo‐γ‐linolenic acid (DGLA) [34]. DGLA serves as a precursor of anti‐inflammatory eicosanoids rather than of pro‐inflammatory arachidonic acid derivatives, a metabolic feature considered central to the cutaneous effects of GLA [34]. Consistent with a role in barrier homeostasis, serum levels of GLA and DGLA are negatively correlated with transepidermal water loss in patients with atopic dermatitis [35]. In cAD, oral administration of BCS oil significantly increased serum levels of GLA and DGLA [36], although clinical improvements in pruritus and skin lesions were limited and did not reach statistical significance [36]. In humans, randomized controlled trials have suggested that BCS oil supplementation can reduce the incidence and severity of atopic dermatitis, particularly when administered during pregnancy and early infancy [37].

Together, these findings indicate that while GLA supplementation may beneficially modulate fatty acid metabolism, its clinical efficacy, especially in canine AD, remains uncertain and warrants further investigation.

Building on this evidence, the present study aimed to investigate whether CBD‐enriched hemp oil, GLA‐enriched BCS oil, and vitamin D_3_, alone or in combination, can exert protective effects in vitro keratinocyte system based on exposure to cytokines associated with canine atopic dermatitis. Specifically, we assessed their impact on basal keratinocyte proliferation, differentiation, barrier integrity, and oxidative stress under inflammatory conditions. By exploring these key cellular mechanisms, our goal was to evaluate the potential of these natural topical formulations as safe, low‐cost adjuvants to complement current therapeutic options for canine atopic dermatitis.

## 2 Materials and Methods

### 2.1 Reagents

Hemp oil and BCS oil were purchased respectively from MH medical hemp GmbH (Wilhelm Kabus Str. 74 D‐10829 Berlin) and NEW INGREDIENTS COMPANY (05018 Orvieto (TR) – ITALIA). Hemp oil was fully profiled for cannabinoid composition. Quantification of major phytocannabinoids revealed 4.22% cannabidiolic acid (CBDA), 6.29% cannabidiol (CBD), 0.08% cannabinol (CBN), 0.04% cannabichromene (CBC), 0.15% Δ^9^‐tetrahydrocannabinolic acid (THCA), and undetectable levels of Δ^9^‐tetrahydrocannabinol (THC). Based on these values, total CBD content was 9.99% w/w and total THC content 0.19% w/w. All oils were stored at 4°C in amber containers and used within the period indicated by the manufacturer. BCS oil was analyzed for key oxidative and compositional parameters, showing a free fatty acid content of 0.2% (expressed as oleic acid; AOCS Ca 5a‐40), an iodine value of 176.9 g I_2_/100 g (AOCS Cd 1‐25), and a peroxide value of 3 meq/kg. The oil contained 17.85% ± 5% γ‐linolenic acid (GLA) relative to total fatty acids, as determined by methods NGD C41‐1976/C42‐1976. Vitamin D3 (cholecalciferol, ≥ 98% purity) was obtained from Sigma‐Aldrich (St. Louis, MO, USA). Canine progenitor epidermal keratinocytes (CPEK) and culture medium kit (CnT‐09) were purchased from CELLnTEC (Bern, Switzerland). Interferon‐γ (IFN‐γ) (Cat: 781‐CG), interleukin‐4 (IL‐4) (Cat: 754‐CL), and interleukin‐13 (IL‐13) (Cat: 5894‐CL) were purchased from R&D Systems Inc. (McKinley Place, MN, USA). Unless otherwise specified, all chemicals were obtained from Sigma‐Aldrich (St. Louis, MO, USA).

### 2.2 Preparation of Compounds

For single treatments, 1 μL of hemp oil or blackcurrant seed oil was initially dissolved in 3 μL of dimethyl sulfoxide (DMSO), and subsequent dilutions were performed in complete culture medium to reach the final concentrations used in the assays. The combined oils stock formulation was prepared at a 5:1 ratio of BCS oil to hemp oil (250 mg BCS to 50 mg hemp oil). To this mixture, vitamin D3 was added at final working concentration of 20 μM. All working dilutions were freshly prepared in complete culture medium, and the final DMSO concentration in cell culture did not exceed 0.1% (v/v), a level considered non‐toxic for keratinocytes.

### 2.3 Cell Culture and AD‐Like Inflammatory Stimuli

CPEK cells were maintained in CnT‐09 Canine Epithelial Proliferation Medium supplemented with L‐Glutamine and 10% fetal bovine serum (FBS) provided with culture medium. Cells were incubated at 37°C, with 5% CO_2_ in a humidified chamber. To establish a cytokine‐induced inflammatory keratinocyte model reflecting key features of canine atopic dermatitis, CPEK cells were pre‐stimulated for 24 h to a cytokine mixture consisting of interferon‐γ (IFN‐γ, 5 ng/mL), interleukin‐4 (IL‐4, 50 ng/mL), and interleukin‐13 (IL‐13, 50 ng/mL) [16]. Twenty‐four hours after the inflammatory stimulation, cells were treated for an additional 24 h with either hemp oil, blackcurrant seed oil, vitamin D3, or their combination.

### 2.4 Cell Viability MTT Assay

CPEK cells were seeded at a density of 1 × 10^4^ cells per well in 96‐well plates and allowed to adhere overnight. For single and combined oils testing, a stock solution was prepared by mixing 1 μL of oil with 3 μL of DMSO. From this stock, a 1:1000 dilution was prepared in complete culture medium, which represented the highest concentration tested. Serial 1:2 dilutions were then performed from this starting point, generating a decreasing concentration range for the assay. For vitamin D3, cells were treated with a concentration range of 100, 80, 40, 20, 10, 5, and 2.5 μM. After 24 h of treatment, cells were incubated with MTT solution (0.5 mg/mL) for 30 min at 37°C. Formazan crystals were solubilized in DMSO, and optical densities (OD) were measured at 570 nm using a Varioskan microplate reader (Thermo Scientific, Waltham, MA, USA). Cell viability was expressed as a percentage relative to untreated control cells.

### 2.5 Trypan Blue Exclusion Test

Cell vitality was further assessed by the Trypan Blue exclusion assay. After inflammatory stimulation and subsequent 24 h treatment with hemp oil, blackcurrant seed oil, vitamin D3, or their combination, CPEK cells were harvested by trypsinization and resuspended in complete culture medium. An equal volume of 0.4% Trypan Blue solution was added to the cell suspension, and viable (unstained) and non‐viable (blue‐stained) cells were counted using a hemacytometer under light microscopy. The percentage of viable cells was calculated as the ratio of unstained to total cells.

### 2.6 Flow Cytometric Analysis of Ki‐67 and NRF2

Flow cytometry analysis was performed on 5 × 10^5^ cells per sample, processed in a V‐bottom, untreated 96‐well plate. Additional wells were included for isotype controls, unstained controls, and single‐stained samples with the Live/Dead dye. Except for the unstained control, all wells were stained with LIVE/DEAD Near‐IR (Invitrogen, Thermo Fisher Scientific, Waltham, MA, USA; #L10119) according to the manufacturer's instructions. Following staining, 100 μL of FACS buffer was added to each well to dilute the dye, and the plate was centrifuged at 300× *g* for 5 min at room temperature (RT). The cells were then washed by resuspending the pellets in 200 μL of FACS buffer, followed by centrifugation under the same conditions. Cells were fixed with 2% paraformaldehyde at RT for 15 min and washed twice. Permeabilization was performed by adding 200 μL of 1× Permeabilization Buffer (Invitrogen, Thermo Fisher Scientific, Waltham, MA, USA; #00‐8333‐56) to each well, centrifuging at 400–600× *g* for 5 min at RT, and discarding the supernatant. This step was repeated, and the pellet was resuspended in the residual volume and adjusted to approximately 100 μL with 1× Permeabilization Buffer.

Ki‐67 immunodetection was carried out by incubating the cells with a mouse anti‐Ki‐67 primary antibody (1:50; Invitrogen, Thermo Fisher Scientific, Waltham, MA, USA; Cat: #14‐5699‐82) and with anti‐rabbit NRF2 (C‐20) antibody (1:50; Santa Cruz Biotechnology, Dallas, TX, USA; Cat: SC‐722) diluted in Permeabilization Buffer, for at least 1 h at +4°C, protected from light. After incubation, 200 μL of 1× Permeabilization Buffer was added, and cells were centrifuged and washed as described above. The secondary antibody incubation was performed for 1 h at RT using an Alexa Fluor 647‐conjugated goat anti‐mouse secondary antibody (Invitrogen, Thermo Fisher Scientific, Waltham, MA, USA) diluted 1:200 in Flow Cytometry Staining Buffer for KI‐67 and Goat anti‐Rabbit PE‐conjugated (Invitrogen, Thermo Fisher Scientific, Waltham, MA, USA) diluted 1:200 in the same staining buffer for NRF2. Finally, cells were resuspended in Flow Cytometry Staining Buffer and analyzed using a CytoFLEX flow cytometer (Beckman Coulter, Chaska, MN, USA). Data were processed with the FlowJo software (Tree Star, Ashland, OR, USA). For each experiment, a minimum of 10,000 events was recorded and analyzed. Gating strategy is reported in Figure S1A,B.

### 2.7 Cell Cycle Analysis

Cells were harvested and washed twice with phosphate‐buffered saline (PBS). Approximately, 1 × 10^6^ cells per sample were fixed by adding ice‐cold 70% ethanol dropwise while gently vortexing to prevent cell aggregation. Samples were kept at −20°C for at least 30 min to allow complete fixation. After fixation, cells were centrifuged and washed twice with PBS to remove residual ethanol. The pellets were resuspended in 0.5 mL of FxCycle PI/RNase Staining Solution (Invitrogen, Thermo Fisher Scientific, Waltham, MA, USA; Cat#F10797) and incubated for 30 min at room temperature in the dark. Samples were analyzed without additional washing using a flow cytometer equipped with a 488 nm laser and a 610/20 or 617/20 nm emission filter. DNA content histograms were used to determine the percentage of cells in *G*_0_/*G*_1_, *S*, and *G*_2_/*M* phases using FlowJo software. Doublets and aggregates were excluded by gating on forward scatter height (FSC‐H) versus area (FSC‐A). Gating strategy is reported in Figure S1C.

### 2.8 RNA Preparation and Real‐Time RT‐PCR Analysis

Total RNA was isolated from CPEK cells using the ReliaPrep RNA Cell Miniprep System (Promega, Madison, WI, USA), according to the manufacturer's instructions. RNA quantity and purity were assessed by spectrophotometry (NanoDrop, Thermo Fisher Scientific), and cDNA was synthesized with the SensiFAST cDNA Synthesis Kit (Bioline, London, UK). Quantitative PCR was performed on a StepOne Real‐Time PCR System (Applied Biosystems, Thermo Fisher Scientific) using TaqMan probes (Applied Biosystems). Assay identification numbers are provided in Table 1. Each sample was run in technical duplicate, and three independent biological experiments were performed for each condition. The mean cycle threshold (Ct) value was computed across technical duplicates for each sample. Relative gene expression was calculated with the 2^−ΔCt^ method, using GAPDH as the endogenous reference gene (ΔCt = Ct[target] − Ct[GAPDH]).

**TABLE 1:** TaqMan gene expression assays.

Gene	Assay ID
*GAPDH*	Cf004419463_gH
*KRT5*	Cf02739817_s1
*KRT10*	Cf03023136_m1
*TGM1*	Cf02690736_m1
*CLDN1*	Cf02713195_u1
*TJP1*	Cf01552709_m1
*NFE2L2*	Cf02665308_g1
*BACH1*	Cf02720806_m1
*HMOX1*	Cf02626655_m1

*Note:* All probes were purchased from Thermo Fisher Scientific (Waltham, MA, USA). Assay IDs refer to inventoried TaqMan Gene Expression Assays spanning exon boundaries to avoid genomic DNA amplification.

### 2.9 Immunofluorescence

For immunofluorescence analysis, CPEK cells were cultured on glass coverslips until confluence, stimulated with cytokines, and subsequently treated with oils and/or vitamin D3 for 24 h. Cells were rinsed with PBS, fixed in 3% paraformaldehyde for 20 min, permeabilized with 0.1% Triton X‐100 for 15 min, and blocked with 5% BSA for 1 h at room temperature. Primary antibodies against ZO‐1 (1:150, Invitrogen, Cat# 33‐9100) and involucrin (1:200, Invitrogen, Cat# MA5‐11803) were applied overnight at 4°C in a dark, humidified chamber. After washing, cells were incubated for 1 h at room temperature with donkey anti‐rabbit Alexa Fluor 568‐conjugated secondary antibody (1:200, Thermo Fisher Scientific, Cat# A10042). Coverslips were mounted using ProLong Gold Antifade Mountant (Thermo Fisher Scientific). Images were acquired with a confocal fluorescence microscope (LSM 510; Zeiss, Germany) using identical acquisition settings (laser power, gain, exposure time) across all experimental groups within each independent experiment. Image quantification was performed in Fiji (ImageJ, NIH, USA). For each marker, several non‐overlapping fields per condition were analyzed (*n* = 6 fields per condition for involucrin; *n* = 5 fields per condition for ZO‐1) from three independent experiments. For involucrin, fluorescence intensity was quantified as the mean gray value of the marker channel within manually defined regions of interest (ROIs) covering the cellular cytoplasm, after subtraction of the local background mean gray value measured in cell‐free areas of the same image. For ZO‐1, intensity was quantified separately in two compartments: cell contours were traced manually based on the ZO‐1 signal, and a peri‐membrane band was defined along the cell boundary using the “Make Band” function in Fiji; the remaining intracellular area within each cell contour was considered the cytoplasmic compartment, and mean gray values were extracted from each compartment after background subtraction. Identical thresholding and segmentation parameters were applied to all images within a given experiment.

### 2.10 Scratch Wound Healing Assay

For wound healing experiments, CPEK cells were seeded in 6‐well plates at a density of 1 × 10^6^ cells/well and cultured until they formed a confluent monolayer. Cells were then exposed to the cytokine cocktail for 24 h. After inflammatory stimulation, a linear scratch was created across the monolayer using a sterile 10 μL pipette tip; detached cells were removed by rinsing with PBS, and cultures were subsequently treated with hemp oil, blackcurrant seed oil, vitamin D_3_, or their combination in serum‐free medium. To minimize the contribution of cell proliferation to apparent wound closure, cytosine arabinoside (Ara‐C) was added at a final concentration of 10 μM to all conditions at the time of scratching. Wound closure was monitored at 0, 4, 18, 24, and 48 h using a digital microscope (PAULA, Leica Microsystems, Wetzlar, Germany); reference marks drawn on the underside of the wells ensured that the same scratch region was imaged at each timepoint, so that each well was followed longitudinally throughout the experiment. The scratch width was quantified using Leica Application Suite software (version 4.2), and the percentage of wound closure at each timepoint was calculated as follows:

$$\%\text{closure}=\left[\left(A_{0}-A_{t}\right)/A_{0}\right]\times 100$$

where *A*_0_ is the scratch area at time 0 and *A*_*t*_ the scratch area at time *t*. For each experimental condition, four independent scratches across three independent experiments were analyzed.

### 2.11 Transepithelial Electrical Resistance Measurement

CPEK cells were seeded at a density of 1.8 × 10^4^ cells per insert on 0.4 μm pore‐size transwell membranes (THINCERT, Greiner Bio‐One S.r.l., Rome, Italy) and cultured in complete medium for 3 days until they formed a fully confluent monolayer; cells were maintained at post‐confluence without active induction of terminal differentiation. Transepithelial electrical resistance (TEER) was monitored using a Millicell ERS‐2 voltohmmeter equipped with chopstick electrodes (Millipore, Burlington, MA, USA), and values were corrected for the resistance of a cell‐free, medium‐only insert. Baseline TEER was measured before any treatment to verify monolayer integrity, and only inserts displaying a baseline TEER above 250 Ω cm^2^ were retained for the experiment. Selected monolayers were then exposed to the cytokine cocktail and TEER was recorded after 24 h of inflammatory stimulation; cells were subsequently treated with hemp oil, BSO, vitamin D_3_, or their combination, and TEER was measured again after additional 24 h of treatment. Results are expressed as percentage change relative to pre‐stimulation baseline, to account for inter‐insert variability in monolayer resistance prior to treatment.

### 2.12 Enzyme‐Linked Immunosorbent Assay (ELISA)

Secreted analytes related to oxidative protein damage and to the inflammatory response were quantified by ELISA on cell‐culture supernatants. Conditioned media were collected at the end of the treatment period (24 h of cytokine stimulation followed by 24 h of treatment), centrifuged at 500× *g* for 10 min at 4°C to remove cellular debris, aliquoted, and stored at −80°C until analysis. Each sample was assayed in duplicate, and three independent biological experiments were performed for each condition. All ELISAs were carried out following the manufacturer's instructions; optical density was read on a Varioskan microplate reader (Thermo Fisher Scientific, Waltham, MA, USA), and analyte concentrations were interpolated from a standard curve generated in parallel on the same plate using four‐parameter logistic (4PL) regression. The commercial kits used in this study are listed in Table 2.

**TABLE 2:** Commercial ELISA kits.

Target	Supplier	Catalogue number	Detection range	Assay format
3‐Nitrotyrosine	Antibodies.com	A74164	1.56–50 ng/mL	Competitive
Interleukin‐8	MyBioSourse.com	MBS700607	0.312–20 ng/mL	Sandwich
Interleukin‐6	MyBioSourse.com	MBS735606	1.0–1000 pg/mL	Competitive
CXCL10	MyBioSourse.com	MBS747479	1.0–1000 pg/mL	Competitive
CXCL9	MyBioSourse.com	MBS2615990	15.6–1000 pg/mL	Sandwich
CCL17	MyBioSourse.com	MBS2884353	15.6–1000 pg/mL	Sandwich

*Note:* ELISA kits used to quantify the analytes measured in CPEK cell‐culture supernatants: 3‐nitrotyrosine, as a readout of nitrosative/oxidative protein damage, and the panel of inflammatory mediators covering the NF‐κB (IL‐8, IL‐6), STAT1 (CXCL9, CXCL10), and STAT6 (CCL17) signaling axes. For each kit, the target analyte, supplier, catalogue number, detection range, and assay format are reported.

### 2.13 Statistical Analysis

Statistical analyses were performed in Python (version 3.12) using the SciPy (1.17) and pingouin (0.5) libraries. Data are presented as mean ± SD, with individual data points shown for all groups. For each variable, the assumption of normality was assessed using the Shapiro–Wilk test. For single‐timepoint readouts, groups were compared by one‐way ANOVA followed by pairwise Welch's *t*‐tests which do not assume equal variances with Šidák correction for multiple comparisons; two separate comparison families were defined per readout, one using CTRL as the reference (to test the effect of the inflammatory stimulus) and one using the Inflamed group as the reference (to test the effect of each treatment). For the wound‐closure and TEER assays, in which the same wells/inserts were followed longitudinally, a mixed two‐way ANOVA was applied, with treatment as the between‐subjects factor and time as the within‐subjects (repeated‐measures) factor, followed by per‐timepoint Welch's *t*‐tests with Šidák correction using Inflamed as the reference. Throughout, # denotes a significant difference versus CTRL and * a significant difference versus Inflamed, with *p* < 0.05 considered statistically significant. Treatments were allocated to experimental replicates in a randomized manner, and imaging and flow‐cytometry acquisitions used identical settings across all groups within each experiment. Image‐based quantifications and flow‐cytometry gating were not performed under blinded conditions, which is acknowledged as a potential source of bias.

## 3 Results

### 3.1 Dose–Response Analysis and Selection of Working Concentration

Preliminary assays were conducted to define the optimal experimental conditions and to evaluate the cytotoxicity of the natural compounds in CPEK cells under non‐inflammatory conditions. MTT dose–response experiments (Figure S2A–D) showed that both hemp oil and BCS oil were non‐toxic across the entire range of dilutions tested. Based on these results, the working concentration was set at the fourth 1:2 dilution following the initial 1:1000 dilution, equivalent to roughly 1:8000 of the original stock. The same dilution was applied to all oil formulations, including the mixture of hemp and BCS, to which vitamin D_3_ was added to reach a final concentration of about 20 μM. For vitamin D_3_ alone, the dose–response analysis yielded an estimated EC_50_ of approximately 2.2 × 10^−5^ M, and 20 μM was chosen as the working concentration.

To further exclude any cytotoxic effects associated with the inflammatory treatment, Trypan Blue exclusion tests were performed after 24 h of cytokine stimulation followed by 24 h of exposure to the selected compound concentrations. As shown in Figure S3, neither the inflammatory challenge nor the subsequent treatment with the natural formulations caused significant cell death, confirming the suitability of the experimental conditions adopted for all analyses.

### 3.2 Vitamin D_3_ and Oil Combination With Vitamin Suppress Keratinocyte Hyperproliferation in an Inflamed CPEK Cells

**FIGURE 1:**
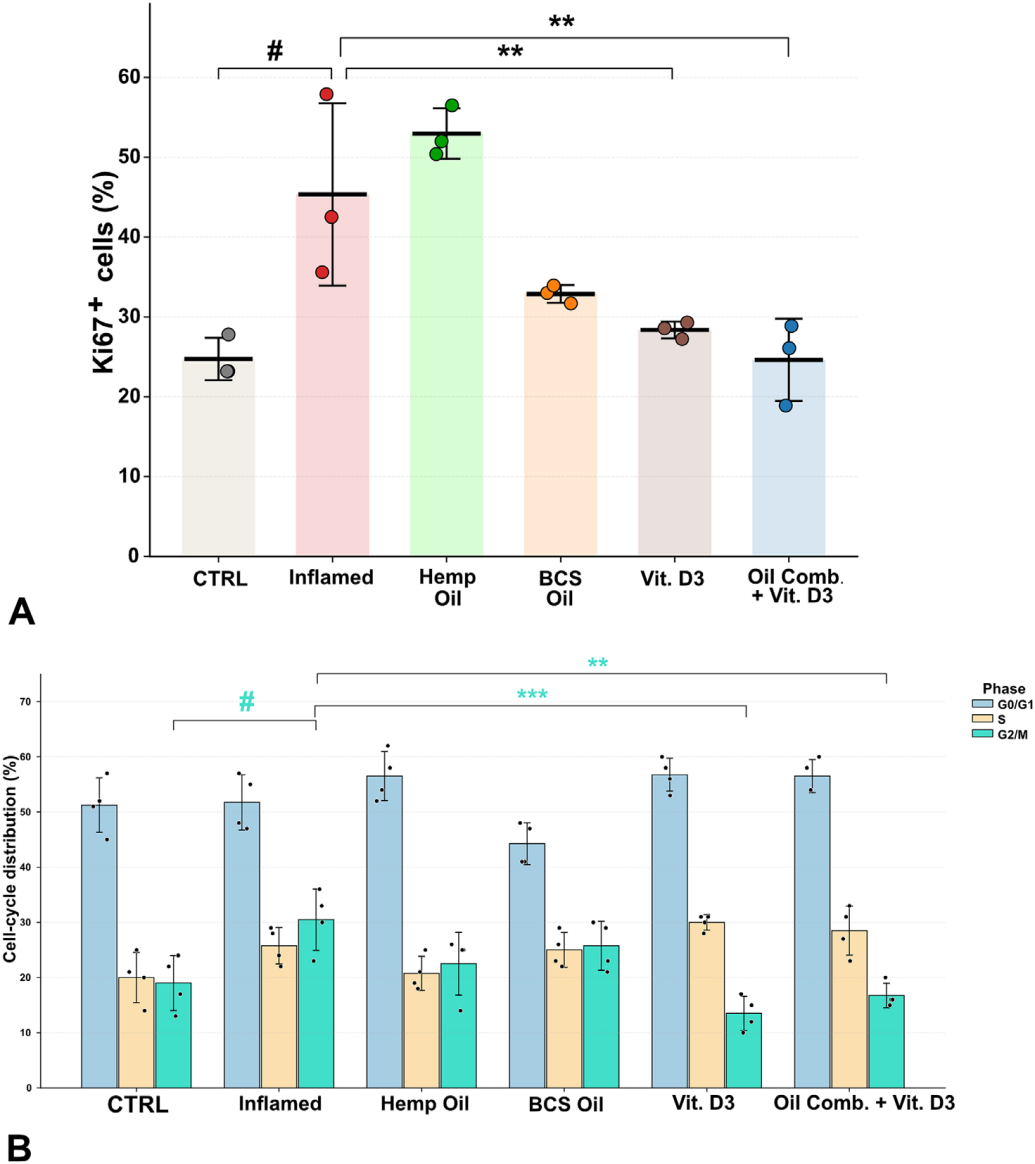
Effects of natural oil formulations and vitamin D_3_ on proliferation and cell cycle distribution of CPEK cells after inflammatory stimulation. (A) Ki67 expression was evaluated by flow cytometry 24 h after the inflammatory stimulus in control cells (CTRL), inflamed (AD), and treated groups (hemp oil, BCS oil, vitamin D_3_, and oil combination + vitamin D_3_). Bars show the mean ± SD (*n* = 3 per group) and individual data points represent each biological replicate. (B) Cell‐cycle phase distribution was analyzed by flow cytometry in the same experimental conditions. Data are shown as mean ± SD; individual data points represent biological replicates. Statistical analysis: One‐way ANOVA followed by pairwise Welch's *t*‐tests with Šidák correction, performed as two independent comparison families per readout—# indicates a significant difference versus CTRL (effect of inflammation) and * indicates a significant difference versus Inflamed (effect of each treatment relative to the inflamed state). #/*p < 0.05; ##/**p < 0.01; ###/***p < 0.001.

Hyperproliferation of keratinocytes is a hallmark of cAD, contributing to epidermal thickening and impaired barrier function [38]. Ki‐67 analysis revealed that inflammatory stimulation induced a marked increase in proliferative keratinocytes (CTRL: 24.7 ± 2.7 vs. Inflamed: 45.3 ± 11.4) (Figure 1A). Incidentally, treatment with hemp oil further enhanced Ki‐67 positivity (53.0 ± 3.2), while BCS exerted a partial but not significant reduction (32.9 ± 1.1). Notably, vitamin D_3_ (28.4 ± 1.1) and the oil combination with vitamin D_3_ (24.6 ± 5.1) effectively suppressed the inflammatory hyperproliferation, restoring levels close to control cells. Cell cycle analysis showed in Figure 1B corroborated these findings. In inflamed keratinocytes, the proportion of cells in G_2_/M phase increased markedly (30.5 ± 5.6) compared to CTRL (19.0 ± 5.0), reflecting accelerated proliferation. Both vitamin D_3_ and the oil combination with vitamin D_3_ reduced the G_2_/M fraction (22.5 ± 3.5 and 22.0 ± 3.2, respectively), with a concomitant shift toward G_0_/G_1_ (CTRL: 51.3 ± 4.9, Inflamed: 51.8 ± 5.0, vitamin D_3_: 56.5 ± 3.0, Oil combination with vitamin D_3_: 56.5 ± 3.0). Together, these data indicate that while hemp oil alone did not attenuate proliferation, vitamin D_3_ and the oil combination with vitamin D_3_ strongly counteracted cytokine‐driven hyperproliferation, normalizing both Ki‐67 expression and cell cycle distribution.

### 3.3 Vitamin D_3_ and Oil Combination Restore Dysregulated Gene Expression of Keratinocyte Differentiation Markers

**FIGURE 2:**
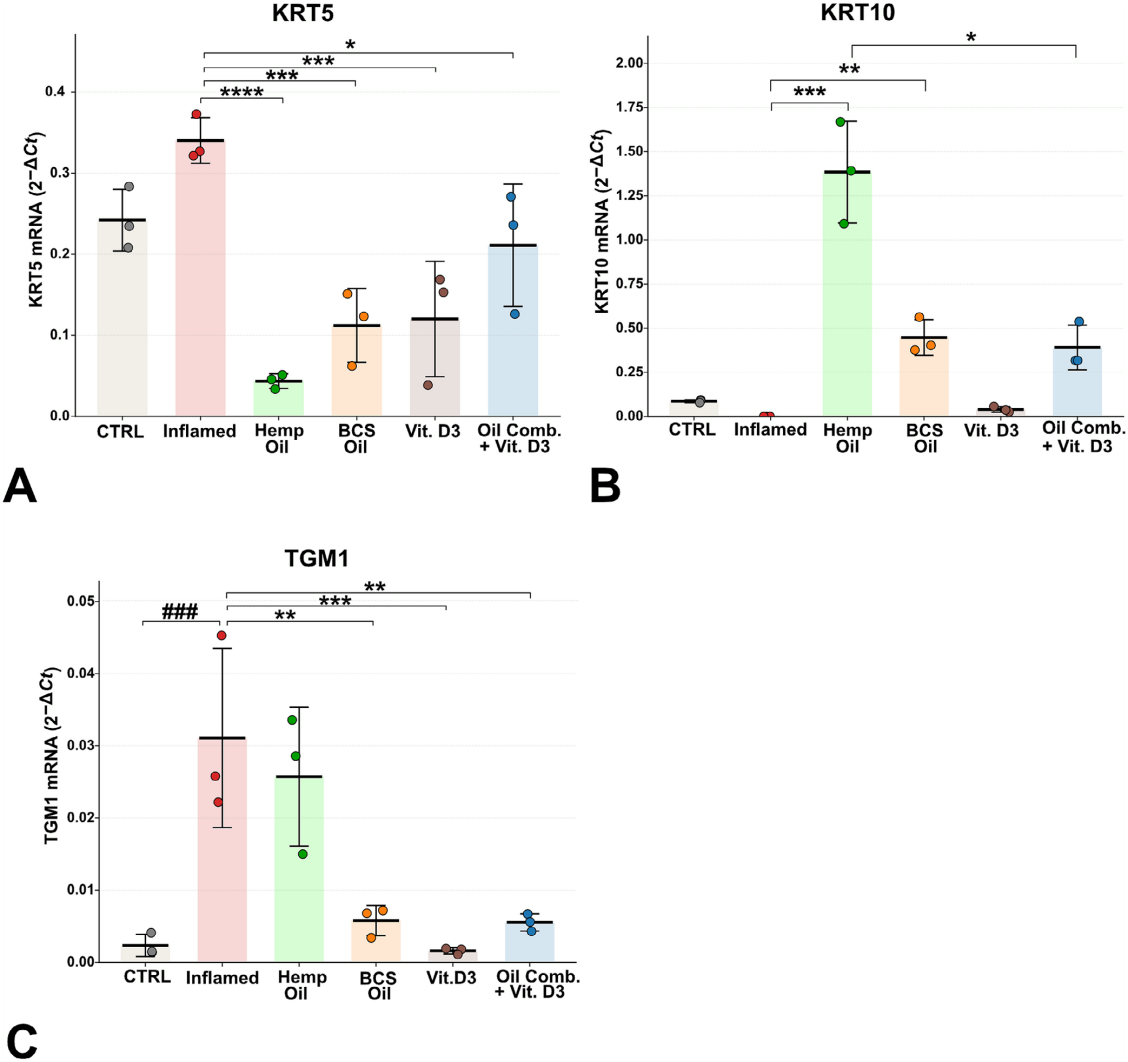
Effects of natural oil formulations and vitamin D_3_ on Keratinocyte Differentiation Marker Expression in CPEK Cells after Inflammatory Stimulation. Keratin 5 (A), Keratin 10 (B), and Transglutaminase 1 (C) expression were evaluated by RT‐PCR 24 h after the inflammatory stimulus in control cells (CTRL), inflamed (cAD), and treated groups (hemp oil, BCS oil, vitamin D_3_, and oil combination + vitamin D_3_). Data are shown as mean ± SD; individual data points represent biological replicates. Statistical analysis: One‐way ANOVA followed by pairwise Welch's *t*‐tests with Šidák correction, performed as two independent comparison families per readout—# indicates a significant difference versus CTRL (effect of inflammation) and * indicates a significant difference versus Inflamed (effect of each treatment relative to the inflamed state). #/*p < 0.05; ##/**p < 0.01; ###/***p < 0.001.

Keratinocyte differentiation is a tightly regulated process essential for maintaining epidermal architecture and barrier function, and its impairment is a hallmark of cAD [10]. To assess the effects of the treatments on epidermal differentiation, we quantified the gene expression of keratin 5 (KRT5), a cytoskeletal protein expressed in proliferative basal keratinocytes, and keratin 10 (KRT10), a marker of early suprabasal differentiation. Inflammatory stimulation significantly altered basal marker expression, with KRT5 markedly increased compared to control (CTRL: 0.242 ± 0.038; Inflamed: 0.340 ± 0.028), indicating sustained basal proliferation under inflammatory conditions. All treatments reduced KRT5 transcript levels relative to the inflamed group, with the most pronounced decrease observed in hemp oil treated cells (0.043 ± 0.009), followed by BCS oil (0.112 ± 0.046), vitamin D_3_ (0.120 ± 0.071), and the oil combination with vitamin D_3_ (0.211 ± 0.076) (Figure 2). In contrast, KRT10, which is typically expressed in suprabasal keratinocytes and inversely correlated with KRT5, was undetectable in inflamed cells, but became clearly detectable in all treatments, suggesting an early activation of the differentiation program rather than restoration of baseline expression. The expression of transglutaminase‐1 (TGM1), an enzyme responsible for cross‐linking structural proteins during cornified envelope assembly, was markedly affected by the inflammatory stimulus. Inflamed keratinocytes displayed a robust induction of TGM1 transcripts compared with control cells (CTRL: 0.0023 ± 0.0015; Inflamed: 0.0311 ± 0.0124), reflecting an abnormal activation of late differentiation pathways under inflammatory stress. Treatment with vitamin D_3_ significantly reduced TGM1 expression (0.0016 ± 0.0004), restoring values close to those of unstimulated controls. A similar downregulatory effect was observed with the oil combination with vitamin D_3_ (0.0055 ± 0.0012) and with blackcurrant seed oil (0.0058 ± 0.0021), whereas hemp oil induced only a partial reduction (0.0257 ± 0.0096) (Figure 2).

### 3.4 Vitamin D_3_, Hemp Oil, and BCS Oil Counteract Inflammation‐Induced Upregulation of Involucrin in CPEK Cells

**FIGURE 3:**
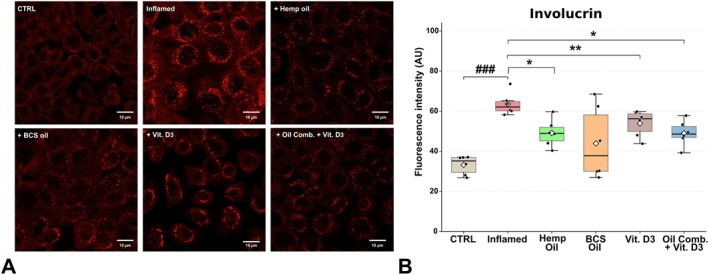
Vitamin D_3_, hemp oil, and BCS oil counteract inflammation‐induced upregulation of involucrin in CPEK cells. (A) Representative confocal microscopy images showing involucrin (IVL) immunofluorescence 24 h after cytokine stimulation in control cells (CTRL), Inflamed, and treated groups (hemp oil, BCS oil, vitamin D_3_, and oil combination + vitamin D_3_). (B) Quantification of involucrin signal intensity from confocal images. Data are shown as mean ± SD; individual data points represent biological replicates. Statistical analysis: One‐way ANOVA followed by pairwise Welch's *t*‐tests with Šidák correction, performed as two independent comparison families per readout—# indicates a significant difference versus CTRL (effect of inflammation) and * indicates a significant difference versus Inflamed (effect of each treatment relative to the inflamed state). #/*p < 0.05; ##/**p < 0.01; ###/***p < 0.001.

Involucrin (IVL) is a major component of the cornified envelope, and a key structural protein required for terminal keratinocyte differentiation and epidermal barrier integrity [39]. In atopic dermatitis (AD), epidermal barrier dysfunction is associated with the dysregulated expression of barrier‐related proteins such as filaggrin, loricrin, and involucrin [13, 14, 15]. Several studies have shown that inflammatory cytokines, particularly IL‐4 and IL‐13, acting through STAT6 and STAT3, downregulate the expression of these differentiation markers, thereby contributing to barrier impairment [15]. Based on this evidence, we investigated whether the inflammatory environment alters involucrin (IVL) expression in basal keratinocytes and whether treatment with hemp oil, BCS oil, and vitamin D_3_ could modulate this effect. Immunofluorescence analysis performed 24 h after cytokine stimulation (Figure 3) revealed a marked increase in IVL signal intensity compared with control cells (INFLAMED: 63.54 ± 5.53 vs. CTRL: 33.18 ± 4.67), indicating premature activation of differentiation‐associated pathways in response to inflammation. Treatment with hemp oil (49.11 ± 6.67), BCS oil (43.87 ± 17.9), vitamin D_3_ (53.86 ± 6.5), or their combination (48.99 ± 6.29) partially normalized involucrin levels. These findings suggest that the treatments, particularly BCS oil and the combined oil + vitamin D_3_ formulation, suggest that these treatments more effectively counteract inflammation‐induced involucrin overexpression and help preserve basal keratinocyte identity.

### 3.5 Hemp Oil Preferentially Modulates Tight‐Junction Markers, With Limited Effects of the Other Treatments on Barrier Resistance

**FIGURE 4:**
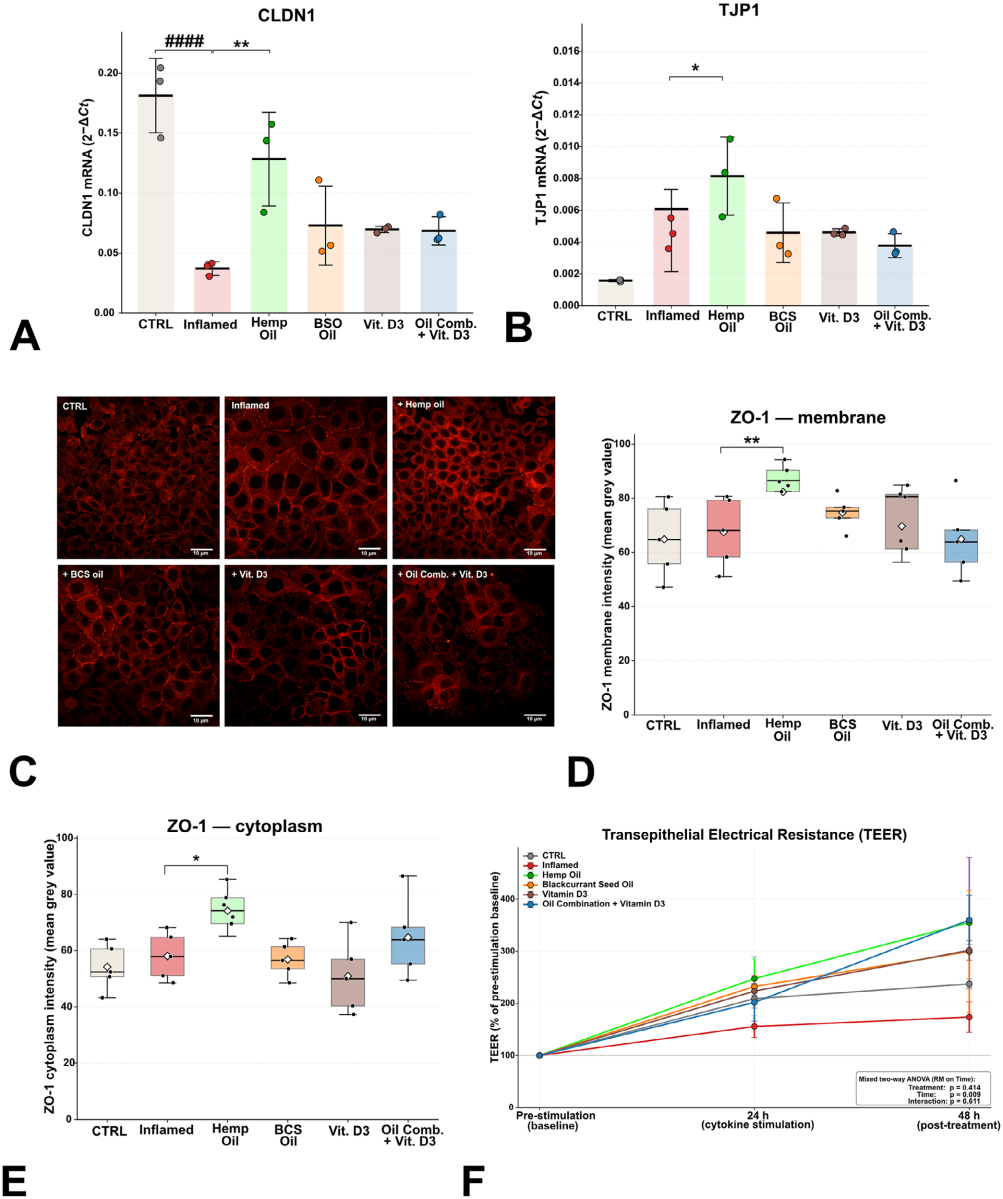
Hemp oil preferentially modulates tight‐junction marker expression in the inflamed CPEK model. (A) Gene expression of claudin‐1 (CLDN1) evaluated by RT‐qPCR 24 h after cytokine stimulation in control cells (CTRL), Inflamed, and treated groups (hemp oil, BCS oil, vitamin D_3_, and oil combination + vitamin D_3_). Data are shown as mean ± SD of 2^−ΔCt^ relative expression (*n* = 3 biological replicates per group, GAPDH as endogenous reference); individual data points represent each biological replicate. (B) Gene expression of tight‐junction protein‐1 (TJP1) evaluated by RT‐qPCR in the same experimental conditions. Data are shown as mean ± SD of 2^−ΔCt^ relative expression (*n* = 3 biological replicates per group, GAPDH as endogenous reference); individual data points represent each biological replicate. (C) Representative confocal microscopy images of ZO‐1 immunofluorescence acquired 24 h after cytokine stimulation in the same experimental groups; nuclei were counterstained with DAPI. Scale bar: 10 μm. (D) Quantification of membrane‐associated ZO‐1 fluorescence intensity, performed in Fiji on confocal images using the “Make Band” tool to define the peri‐membrane region (*n* = 5 non‐overlapping fields per condition, from three independent experiments). Box‐and‐scatter plots show individual measurements; the box represents the interquartile range, the horizontal line within each box the median, the white diamond the mean, and whiskers extend to the most extreme data points within 1.5 × IQR. (E) Quantification of cytoplasmic ZO‐1 fluorescence intensity, performed in Fiji on confocal images by defining the intracellular region within the cell contour, excluding the peri‐membrane band (*n* = 5 non‐overlapping fields per condition, from three independent experiments). (F) Transepithelial electrical resistance (TEER) measured as a functional readout of paracellular barrier integrity. Each insert was followed longitudinally so that the same monolayer was measured at pre‐stimulation (baseline), at 24 h (post‐cytokine stimulation) and at 48 h (post‐treatment). Data are expressed as percentage of the per‐insert pre‐stimulation baseline, mean ± SEM (*n* = 3 inserts per group). Statistical analysis for panels (A, B, D, and E): One‐way ANOVA followed by pairwise Welch's *t*‐tests with Šidák correction, performed as two independent comparison families per readout—# indicates a significant difference versus CTRL (effect of inflammation) and * indicates a significant difference versus Inflamed (effect of each treatment relative to the inflamed state). #/*p < 0.05; ##/**p < 0.01; ###/***p < 0.001. Statistical analysis for panel (F): Mixed two‐way ANOVA with treatment as the between‐subjects factor and time as the within‐subjects (repeated‐measures) factor, followed by per‐timepoint Welch's *t*‐tests with Šidák correction using Inflamed as the reference (). #/*p* < 0.05; ##/**p < 0.01; ###/***p < 0.001.

Tight junction proteins are essential components of the epidermal barrier, and their dysregulation represents a key pathogenic feature of cAD [8, 40]. Consistent with previous evidence showing that Th2 cytokines downregulate claudin‐1 (*CLDN1*) and alter tight junction protein‐1 (*TJP1*/ZO‐1) signaling through ERK‐, STAT3‐, and STAT6‐dependent pathways [41, 42]; inflammatory stimulation of CPEK cells modified the transcriptional profile of these genes (Figure 4A,B). Exposure to pro‐inflammatory cytokines caused a strong downregulation of *CLDN1* mRNA (Inflamed: 0.037 ± 0.005 vs. CTRL: 0.181 ± 0.031), confirming barrier‐related gene suppression. Among the tested natural compounds, hemp oil induced the most pronounced recovery of *CLDN1* expression (0.128 ± 0.039). In contrast, *TJP1* transcription was moderately increased in inflamed cells (Inflamed: 0.004 ± 0.000 vs. CTRL: 0.001 ± 0.000), suggesting a compensatory response to tight junction disruption. Hemp oil further enhanced *TJP1* expression (0.008 ± 0.002), while the other treatments did not significantly modify the levels observed under inflammatory conditions. To assess whether transcriptional modulation of *TJP1* was accompanied by changes in ZO‐1 abundance and subcellular distribution, fluorescence intensity was quantified separately along the plasma membrane and within the cytoplasmic compartment. In the membrane‐associated fraction, ZO‐1 signal modestly increased under inflammatory conditions (CTRL: 58.90 ± 7.69; Inflamed: 72.22 ± 7.23) and was further elevated following hemp oil treatment (89.64 ± 4.01) (Figure 4C,D). A similar trend was observed in the cytoplasmic compartment (Figure 4C,E), where fluorescence intensity showed a non‐significant increase from control to inflamed cells (CTRL: 52.21 ± 6.25; Inflamed: 62.10 ± 4.55), and a marked enhancement after hemp oil exposure (79.36 ± 3.74) (Figure 4C–E). These data indicate that inflammation increases overall ZO‐1 signal intensity, and hemp oil amplifies this effect, consistent with its transcriptional upregulation of TJP1. To determine whether these molecular and distributional changes translated into functional improvements of the epithelial barrier, transepithelial electrical resistance (TEER) was evaluated. Inflammatory stimulation attenuated the time‐dependent increase in TEER observed in unstimulated monolayers: at 24 h, inflamed cells reached 156% ±37% of their pre‐stimulation baseline compared with 209% ± 31% in CTRL (Welch's *t*‐test, *p* = 0.13) (Figure 4F). At 48 h, all treatments tended to enhance TEER recovery above the inflamed condition, with vitamin D_3_ (302% ± 33%), the oil combination (360% ± 82%), hemp oil (355% ± 216%), and BCS oil (299%± 204%) all exceeding the inflamed value (174% ± 51%) (Figure 4F); however, none of these comparisons reached statistical significance. A mixed two‐way ANOVA on the post‐stimulation timepoints revealed a significant effect of Time (*p* = 0.009), but no significant effect of Treatment (*p* = 0.41) or Treatment × Time interaction (*p* = 0.61), suggesting that the trends observed are coherent but underpowered at the current sample size. Collectively, these findings demonstrate that hemp oil exerts the strongest protective effect on tight junction integrity in CPEK cells, enhancing CLDN1 and TJP1/ZO‐1 expression and promoting functional recovery of the epithelial barrier.

### 3.6 All Treatments Improve Early Keratinocyte Migration, but Only Hemp Oil Sustains Accelerated Scratch Closure Under Inflammatory Conditions

**FIGURE 5:**
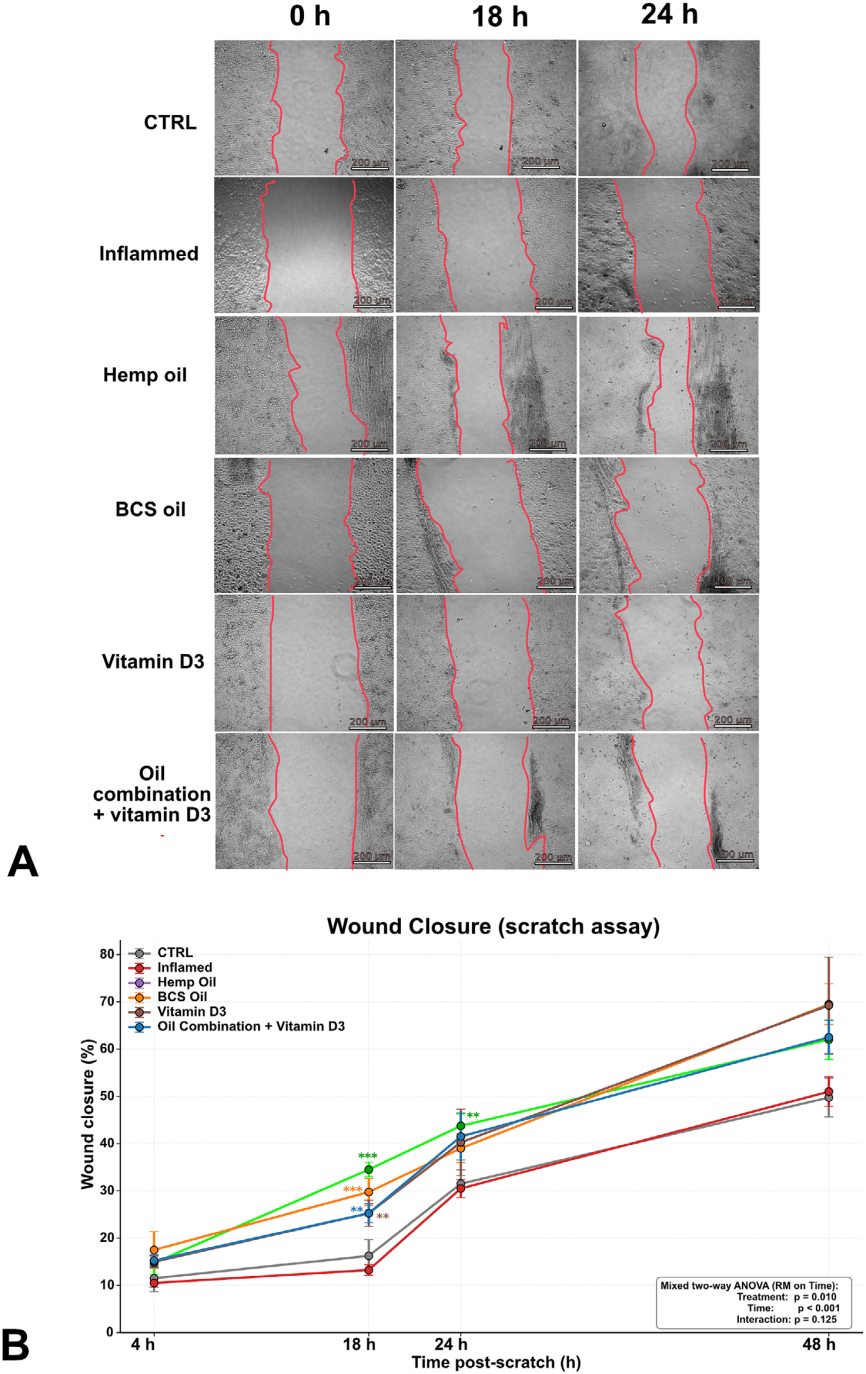
Effects of natural oil formulations and vitamin D_3_ on keratinocyte migration in an inflammatory scratch assay. Keratinocyte migration was assessed using a scratch assay performed in the presence of Ara‐C to suppress proliferation and isolate migration‐driven wound closure. (A) Representative phase‐contrast images of scratch wounds at 0, 18, and 24 h post‐scratch in control (CTRL), Inflamed, and treated groups (hemp oil, BCS oil, vitamin D_3_, and oil combination + vitamin D_3_). Yellow lines indicate the manually traced wound margins. Representative images at 0, 18, and 24 h are shown, corresponding to baseline and to the timepoints displaying the most pronounced between‐group differences in the quantitative analysis; the complete time course (0, 4, 18, 24, and 48 h) is reported in panel (B). Scale bar: 200 μm. (B) Quantification of wound closure (%) at 4, 18, 24, and 48 h. Data are presented as mean ± SEM (*n* = 4 independent scratches per condition, across three independent experiments). Statistical analysis: Mixed two‐way ANOVA with treatment as the between‐subjects factor and time as the within‐subjects (repeated‐measures) factor, followed by per‐timepoint Welch's *t*‐tests with Šidák correction using Inflamed as the reference. *#/p* < 0.05; ##/**p < 0.01; ###/***p < 0.001.

Keratinocyte migration plays a central role in epidermal repair and is strongly impaired in atopic dermatitis (AD), where pruritus‐associated mechanical injury and type‐2 cytokine–driven inflammation delay wound closure and contribute to barrier dysfunction [43]. Inefficient wound healing in this inflammatory context can promote dysregulated tissue remodeling, favoring excessive fibroblast activation, aberrant collagen deposition, and the development of hypertrophic or keloid‐like scarring in predisposed individuals [44]. To specifically assess the migratory capacity of CPEK cells under inflammatory conditions and in response to natural compounds, a scratch assay was performed in the presence of 10 μM cytosine arabinoside (Ara‐C), which was used to suppress proliferation and allow evaluation of migration‐dependent wound closure. At early time points (4 h), no significant differences were detected among the experimental conditions, indicating comparable initial motility across groups. Divergence became evident at 18 h (Figure 5), when untreated inflamed cells exhibited reduced wound closure (13.25% ± 2.21%) compared with control CPEK cells (16.25% ± 6.80%). All treatments accelerated wound healing at this time point, with hemp oil showing the strongest effect (34.50% ± 3.10%), followed by blackcurrant seed oil (29.75% ± 5.70%), vitamin D_3_ (25.20% ± 5.50%), and the oil combination with vitamin D_3_ (25.25% ± 4.00%) (Figure 5). At 24 h, hemp oil remained the only treatment producing a significant enhancement of wound closure (62.00% ± 8.40%), markedly outperforming both inflamed (30.50% ± 3.80%) and control cells (31.50% ± 5.90%) (Figure 5). By 48 h, no significant differences persisted among groups, consistent with the near‐complete closure of the scratch in all conditions and the intrinsic limitations of the assay at late time points (Figure 5). Overall, these data indicate that inflammation slows keratinocyte migration in this canine AD model, and that natural compounds can partially restore motility, with hemp oil exerting the most pronounced and sustained pro‐migratory activity.

### 3.7 Cytokine Stimulation Triggers Nitrosative Stress and Engages the NRF2/BACH1/HMOX1 Axis, Which Is Differentially Modulated by the Four Treatments

**FIGURE 6:**
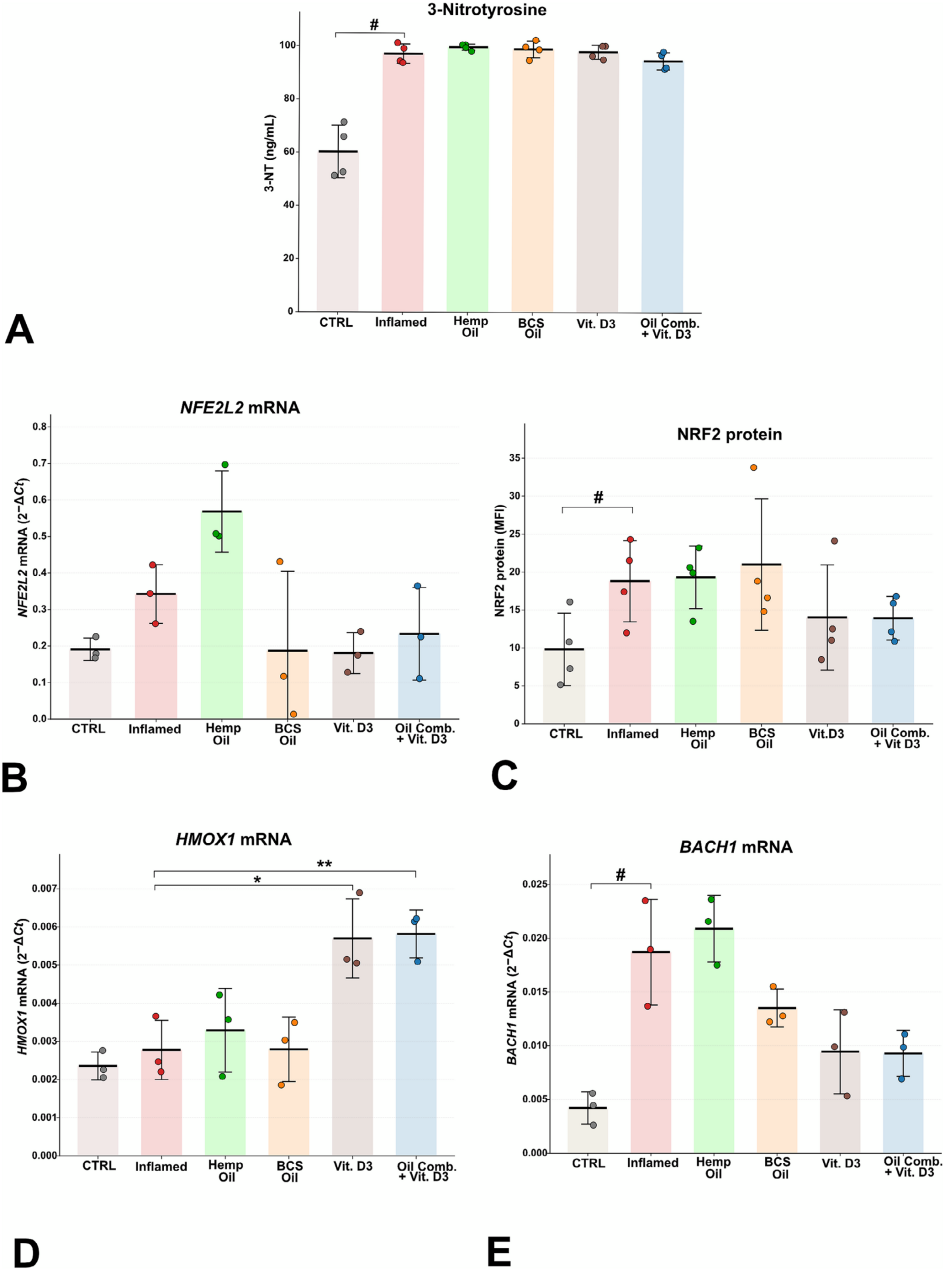
Inflammation induces sustained nitrosative stress and engages the NRF2/BACH1/HMOX1 axis, which is differentially modulated by the four treatments. (A) 3‐Nitrotyrosine (3‐NT) concentration in cell‐culture supernatants, quantified by sandwich ELISA and concentrations obtained by 4‐parameter logistic interpolation of the standard curve. Data are shown as mean ± SD with individual biological replicates (*n* = 4 per group). (B) *NFE2L2*, (D) *HMOX1*, and (E) *BACH1* mRNA expression assessed by RT‐qPCR. Data are shown as mean ± SD of 2^−ΔCt^ relative expression (*n* = 3 biological replicates per group, *GAPDH* as endogenous reference); individual data points represent each biological replicate. (C) NRF2 protein levels quantified by flow cytometry in fixed and permeabilized CPEK cells, expressed as median fluorescence intensity (MFI). Data are shown as mean ± SD with individual biological replicates (*n* = 4 per group). Statistical analysis: One‐way ANOVA followed by pairwise Welch's *t*‐tests with Šidák correction, performed as two independent comparison families per readout—# indicates a significant difference versus CTRL (effect of inflammation) and * indicates a significant difference versus Inflamed (effect of each treatment relative to the inflamed state). #/*p < 0.05; ##/**p < 0.01; ###/***p < 0.001.

Oxidative stress is a well‐recognized contributor to barrier dysfunction in atopic dermatitis (AD), where type‐2 cytokines and chronic mechanical injury increase ROS and weaken tight junction organization [45, 46]. To validate the relevance of our in vitro model in this respect, we first quantified 3‐nitrotyrosine (3‐NT), a stable footprint of peroxynitrite‐mediated protein nitration [47]. 3‐NT levels were strongly increased in inflamed cells compared to CTRL (97.1 ± 3.5 vs. 60.3 ± 9.5 ng/mL), confirming that the cytokine cocktail elicited a robust nitrosative stress response (Figure 6A). None of the treatments reduced 3‐NT relative to the inflamed condition (range 94.7–99.7 ng/mL; all comparisons ns vs. Inflamed). This is consistent with the cumulative nature of the marker: protein nitration occurring during the initial 24 h of cytokine exposure remains in the protein pool sampled at 48 h, and the treatment window (24 h) is shorter than the typical turnover of nitrated proteins. Given this evidence of sustained oxidative challenge, we next interrogated the KEAP1–NRF2 axis, the master regulator of the cytoprotective antioxidant response [48, 49, 50, 51]. At the transcript level, *NFE2L2* mRNA tended to be upregulated in inflamed cells compared with CTRL (0.34 ± 0.08 vs. 0.19 ± 0.03), although this trend did not reach statistical significance (Figure 6B). Hemp oil produced a marked further induction of *NFE2L2* mRNA, reaching levels significantly higher than CTRL (0.57 ± 0.11; # vs CTRL), in keeping with previous reports of cannabidiol‐driven NRF2 activation in keratinocytes [31]; blackcurrant seed oil, vitamin D_3_ and the combined formulation showed a trend of decline toward control levels, without reaching statistical significance. At the protein level, NRF2 was elevated in inflamed cells compared with CTRL (18.8 ± 5.4 vs. 9.8 ± 4.9 MFI; # vs. CTRL; Figure 6C); hemp oil maintained NRF2 protein at levels comparable to the inflamed condition, whereas blackcurrant seed oil, vitamin D_3_ and the combined formulation showed intermediate values without reaching statistical significance.

To assess whether the engagement of NRF2 was reflected in the downstream transcriptional output, we measured *HMOX1*, the prototypical NRF2 target gene, and *BACH1*, a transcriptional repressor that competes with NRF2 for binding to antioxidant response elements on shared target genes including *HMOX1* [31, 48]. *HMOX1* mRNA did not differ significantly between inflamed and control cells (Figure 6D), consistent with the well‐described early‐peak/late‐decline kinetics of this immediate‐early gene, which has typically returned toward basal levels by 24–48 h after stimulation. In contrast, *BACH1* mRNA was markedly upregulated by inflammation (0.019 ± 0.005 vs. 0.004 ± 0.001; # vs. CTRL; Figure 6E). The four treatments differentially modulated this axis: hemp oil and blackcurrant seed oil did not significantly affect either *HMOX1* or *BACH1* mRNA expression relative to the inflamed condition, whereas vitamin D_3_ and the combined formulation produced a coordinated rebalancing of the axis—vitamin D_3_ significantly increased *HMOX1* expression (* vs. Inflamed; Figure 6D), the combined formulation significantly increased *HMOX1* (** vs. Inflamed) and concurrently reduced *BACH1* expression (* vs. Inflamed; Figure 6E). Taken together, these results provide a coherent description of the redox state of the inflamed CPEK model at the 48‐h timepoint: the cytokine cocktail elicits a sustained nitrosative stress (3‐NT) that is not reversed by any of the treatments consistent with the cumulative nature of protein nitration over the experimental window and engages the NRF2 transcriptional axis at both the mRNA and protein levels, while concurrently inducing the transcriptional repressor BACH1. The four treatments exhibit a clearly differential effect on this axis: hemp oil and blackcurrant seed oil do not modulate the NRF2/BACH1/HMOX1 pattern at the timepoint assessed, whereas vitamin D_3_ and especially the combined formulation rebalance the axis by relieving BACH1‐mediated repression and increasing HMOX1 expression, with the combined formulation also producing a coordinated normalization of NRF2 protein levels.

### 3.8 Inflammatory Mediator Secretion Confirms the Engagement of STAT1 and NF‐κB Signaling Axes by the Cytokine Cocktail and Reveals a Pathway‐Specific Modulation by the Tested Compounds

**FIGURE 7:**
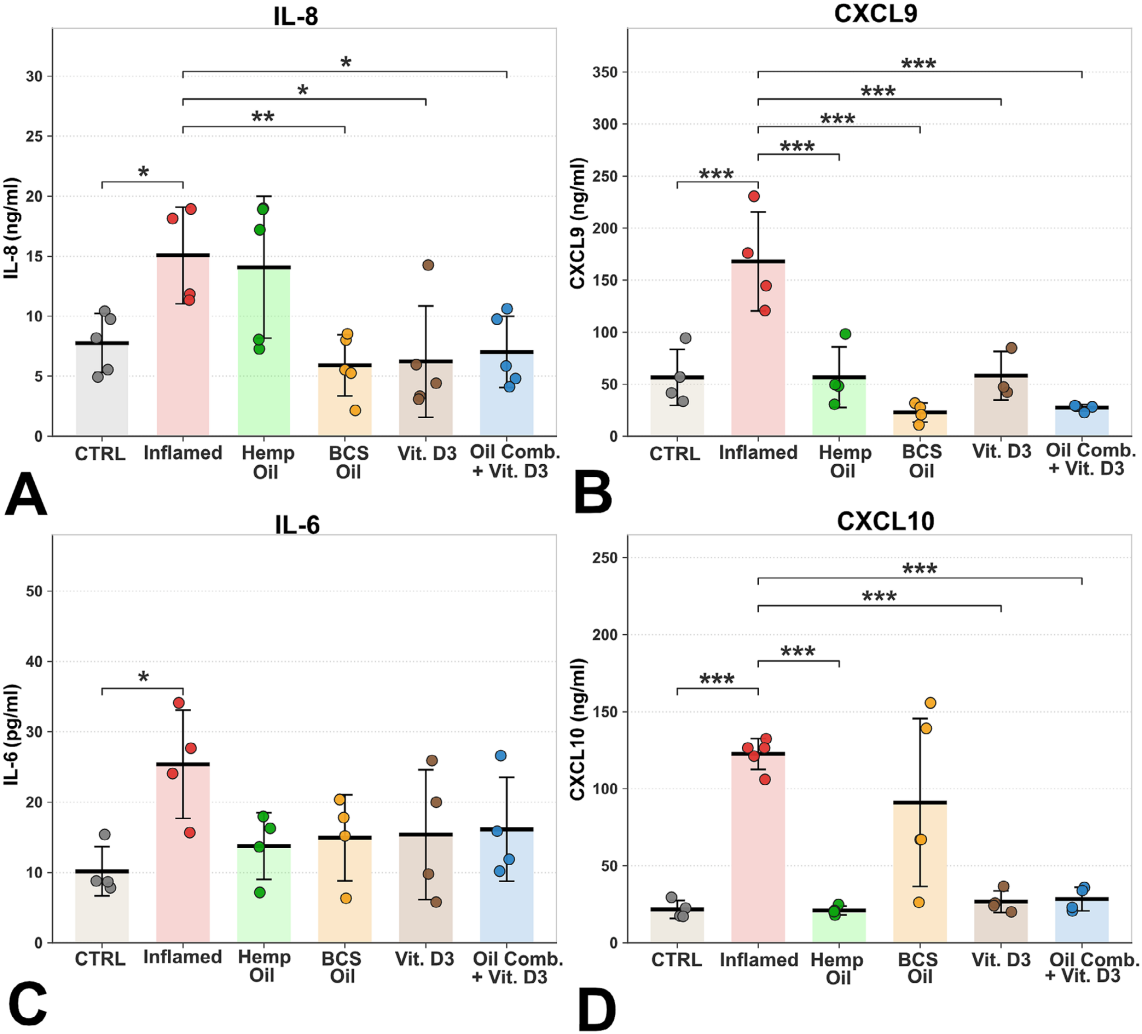
Secreted inflammatory mediator profile reveals a pathway‐specific footprint of the four treatments on STAT1‐ and NF‐κB‐driven signaling. Cytokine and chemokine concentrations were quantified by sandwich ELISA in cell‐culture supernatants from control (CTRL), Inflamed, and treated groups (hemp oil, blackcurrant seed oil, vitamin D_3_, and oil combination + vitamin D_3_), collected at the end of the 48‐h experimental protocol (24 h cytokine stimulation followed by 24 h treatment). (A) IL‐8 (CXCL8) and (C) IL‐6, two NF‐κB‐dependent inflammatory cytokines, used as functional readouts of the NF‐κB signaling axis (IL‐6 also acting as a downstream amplifier of STAT3 through its known autocrine loop in keratinocytes). (B) CXCL9 and (D) CXCL10, two IFN‐γ‐induced, STAT1‐dependent chemokines, used as functional readouts of the STAT1 signaling axis. A fifth mediator, CCL17/TARC, was also measured in the same supernatants as a readout of the IL‐4/IL‐13/STAT6 axis but was below the lower limit of quantification of the assay in all samples, including the inflamed condition, and is therefore not displayed. Data are shown as mean ± SD; individual data points represent each biological replicate. Sample sizes per panel: (A) IL‐8 *n* = 6 (CTRL, hemp oil, BCS oil, vitamin D_3_, combo), *n* = 5 (Inflamed); (B) CXCL9 *n* = 5 (CTRL, inflamed, hemp oil, BCS oil, combo), *n* = 4 (vitamin D_3_); (C) IL‐6 *n* = 5 per group; (D) CXCL10 *n* = 5 (CTRL, hemp oil, vitamin D_3_, combo), *n* = 6 (Inflamed, BCS oil). Statistical analysis: One‐way ANOVA followed by pairwise Welch's *t*‐tests with Šidák correction, performed as two independent comparison families per readout—# indicates a significant difference versus CTRL (effect of inflammation) and * indicates a significant difference versus Inflamed (effect of each treatment relative to the inflamed state). #/*p < 0.05; ##/**p < 0.01; ###/***p < 0.001.

To characterize the inflammatory state of the model and to assess the functional engagement of the major signaling axes activated by the cytokine cocktail, we quantified five cytokines and chemokines in cell‐culture supernatants by ELISA: IL‐8 (CXCL8) and IL‐6 as readouts of NF‐κB‐driven inflammatory signaling, with IL‐6 also functioning as a downstream amplifier of STAT3 through its known autocrine loop; CXCL9 and CXCL10 as IFN‐γ/STAT1‐specific readouts; and CCL17/TARC as a readout of the IL‐4/IL‐13/STAT6 axis. The cytokine cocktail induced a significant secretion of IL‐8 by CPEK keratinocytes (15.1 ± 3.5 vs. 7.8 ± 2.2 ng/mL in CTRL; # vs. CTRL; Figure 7A), confirming the engagement of the NF‐κB inflammatory branch. The four treatments differentially modulated IL‐8 secretion: blackcurrant seed oil, vitamin D_3_ and the combined formulation significantly reduced IL‐8 relative to the inflamed condition (BCS **, Vit D_3_ *, Combo *), whereas hemp oil did not. Engagement of the IFN‐γ/STAT1 axis was confirmed by a marked, three‐fold induction of CXCL9 secretion in inflamed cells compared to CTRL (168.0 ± 41.1 vs. 56.6 ± 23.3 ng/mL; # vs. CTRL; Figure 7B). All four treatments significantly reduced CXCL9 relative to the inflamed condition (Hemp Oil **, BCS **, Vit D_3_ **, Combo **). A second NF‐κB‐dependent cytokine, IL‐6, was also significantly induced by inflammation (25.4 ± 6.7 vs. 10.2 ± 3.0 pg/mL in CTRL; # vs. CTRL; Figure 7C); all treatments modestly reduced IL‐6 secretion, but the differences did not reach statistical significance. The induction of the STAT1 axis was further confirmed by an approximately six‐fold increase in CXCL10 secretion in inflamed cells (122.5 ± 9.0 vs. 21.7 ± 5.0 ng/mL; #*** vs. CTRL; Figure 7D). Hemp oil, vitamin D_3_ and the combined formulation produced a near‐complete normalization of CXCL10 secretion (all *** vs. Inflamed), whereas blackcurrant seed oil did not significantly reduce CXCL10 levels. CCL17/TARC, included as a readout of the IL‐4/IL‐13/STAT6 axis, was below the lower limit of quantification of the assay in all conditions, including the inflamed group, and the corresponding data are therefore not shown.

Taken together, these results document a robust engagement of both the NF‐κB and the STAT1 signaling axes in inflamed CPEK keratinocytes with a particularly marked induction of the IFN‐γ/STAT1‐specific chemokines CXCL9 and CXCL10 and reveal a clearly differential pharmacological footprint of the four treatments across these two axes. Hemp oil acts preferentially on the STAT1 chemokine arm (significant reduction of CXCL9 and near‐complete normalization of CXCL10) without significantly affecting NF‐κB‐dependent cytokines. Blackcurrant seed oil acts preferentially on the NF‐κB‐dependent IL‐8 (significant reduction) without normalizing the STAT1‐driven CXCL10. Vitamin D_3_ and the combined formulation display the broadest action, with significant effects across both signaling axes (CXCL9, CXCL10, IL‐8).

## 4 Discussion

This study provides an integrated analysis of the effects of hemp oil, blackcurrant seed oil, vitamin D_3_, and their combination on cytokine‐stimulated canine progenitor epidermal keratinocytes (CPEK), used here as an acute in vitro model of canine atopic dermatitis (cAD). By examining multiple interconnected features—proliferation, differentiation, tight‐junction organization, barrier function, migration, oxidative stress, and cytokine secretion—we delineate the *differential* signatures of these four natural formulations under inflammatory conditions: hemp oil acts preferentially on tight‐junction integrity, NRF2 activation, and the STAT1 chemokine arm; BCS oil on NF‐κB‐dependent IL‐8 secretion and wound‐closure dynamics; vitamin D_3_ on proliferation and on the downstream NRF2/HMOX1/BACH1 transcriptional output; the combined formulation cumulates the broadest action across these axes.

Keratinocyte hyperproliferation is a defining feature of both human AD and cAD, driven by chronic exposure to type‐2 cytokines (IL‐4; IL‐13) and mechanical injury associated with pruritus [52, 53]. In line with this, cytokine stimulation in our model increased Ki‐67 expression and expanded the G_2_/M cell‐cycle fraction, reflecting accelerated keratinocyte cycling. Vitamin D_3_, both alone and in the combined formulation, significantly attenuated this hyperproliferative response, restoring proliferation indices and cell‐cycle distribution toward control levels. These findings are consistent with the established role of vitamin D_3_ in promoting G_0_/G_1_ arrest and coordinating the transition from proliferation to differentiation in keratinocytes [54, 55]. By contrast, hemp oil did not suppress cytokine‐driven proliferation and was associated with a further increase in Ki‐67 positivity, indicating that its biological activity in this context is not primarily directed toward growth restraint. This observation is consistent with previous evidence [31] that cannabidiol, the major bioactive constituent of hemp oil, directly stimulates keratinocyte proliferation in vivo, with increased epidermal thickness and upregulation of hyperproliferation and wound repair. This divergence underscores that distinct natural compounds may target different facets of keratinocyte dysregulation in cAD and highlights vitamin D_3_ as the most effective modulator of proliferation in this model.

Inflammation‐induced disruption of keratinocyte differentiation represents another central component of cAD pathophysiology [10, 56]. In our model, cytokine stimulation increased expression of the basal marker KRT5, abolished detectable KRT10, and aberrantly induced late differentiation markers such as TGM1 and involucrin in basal keratinocytes, indicative of a distorted differentiation program. This pattern is partly divergent from the canonical signature of human AD lesional skin and of canine atopic biopsies, where *TGM1* is typically reduced rather than increased, and likely reflects a specific feature of our experimental system. IFN‐γ, present in the cytokine cocktail, is a well‐characterized inducer of squamous differentiation in primary keratinocytes, driving *TGM1* upregulation in parallel with downregulation of cell‐cycle and basal‐compartment genes [57]; at the 48‐h readout, this pro‐differentiative effect is consistent with the increased *TGM1* and the reduced *KRT5* and *KRT10* observed in inflamed cells. All treatments partially corrected this imbalance, as reflected by reduced KRT5 expression and re‐emergence of KRT10 transcripts, suggesting reactivation of early suprabasal differentiation. Vitamin D_3_ and the oil combination were particularly effective in normalizing TGM1 gene expression, while BCS oil also exerted a significant attenuating effect. Hemp oil produced a more limited correction of late differentiation markers, suggesting that its principal effects operate through mechanisms other than direct modulation of the differentiation program.

The same pattern was confirmed at the level of involucrin (*IVL*), a precursor of the cornified envelope and a marker of late keratinocyte differentiation. *IVL* was significantly upregulated in inflamed cells compared with control, a direction that is opposite to what is consistently reported in human AD lesional skin and in canine atopic biopsies, where *IVL* is typically downregulated through the IL‐4/IL‐13–JAK–STAT6/STAT3 axis [15, 58, 59]. As discussed above for *TGM1*, this suppressive effect of the Th2 component of the cocktail typically requires longer cytokine exposures or three‐dimensional culture systems to fully emerge, and is therefore unlikely to dominate in the acute, monolayer setting used here. The four treatments modulated *IVL* in a pattern broadly consistent with the one described for *TGM1*: vitamin D_3_ and the combined formulation reduced its expression toward control values, hemp oil maintained an inflamed‐like profile, and BCS oil produced an intermediate response. Taken together, the parallel modulation of *TGM1* and *IVL* indicates that vitamin D_3_‐containing formulations exert a coordinated restraint of the squamous‐differentiation program engaged in our acute model.

Disruption of tight junctions and impaired epidermal barrier function are key pathogenic features of cAD [1, 6, 11]. Consistent with previous reports [1, 32], cytokine stimulation markedly downregulated claudin‐1 expression, reproducing one of the canonical hallmarks of atopic skin and providing indirect evidence for the engagement of the IL‐4/IL‐13–JAK–STAT6 axis [15]. In contrast, TJP1 mRNA and membrane‐associated ZO‐1 protein were not significantly modified, while the apparent increase in cytoplasmic ZO‐1 staining should be interpreted with caution. Cytokine‐stimulated CPEK cells exhibit a hypertrophic morphology, consistent with the well‐documented cell‐enlargement effect of cytokines acting through STAT3, most notably IL‐6, which is produced autocrinously by inflamed keratinocytes, described by Niehues and colleagues as a hypertrophy‐inducing input on keratinocytes independent of hyperproliferation [60]. The increased per‐cell cytoplasmic ZO‐1 signal measured by immunofluorescence in inflamed cells is therefore most parsimoniously explained as a morphometric consequence of this cellular enlargement, rather than as a genuine increase in the intracellular pool of ZO‐1 protein, an interpretation supported by the unchanged TJP1 mRNA levels, which indicate no transcriptional upregulation of the gene. Two aspects of the tight‐junction readout in our model deserve a specific note: the cytoplasmic rather than membranous accumulation of ZO‐1, and the absence of significant TEER changes. Marsella and colleagues have shown that CPEK cells grown in monolayer display, already at baseline, a predominantly cytoplasmic localisation of ZO‐1 and a markedly lower trans‐epithelial electrical resistance (~150 Ohms/cm) than primary canine keratinocytes from skin biopsies (~2000 Ohms/cm), concluding that monolayer CPEKs are not fully representative of normal canine keratinocytes for permeability studies [61]. The further increase in cytoplasmic ZO‐1 signal observed in inflamed cells therefore overlays a baseline already shifted toward this distribution and is compatible with the cytokine‐induced cellular hypertrophy discussed above, while the unchanged TEER reflects the intrinsically narrow dynamic range of this readout in CPEK monolayers.

Keratinocyte migration is tightly linked to epidermal repair and is often impaired in atopic dermatitis [62]. By performing scratch assays under inflammatory conditions while pharmacologically suppressing proliferation, we specifically assessed migration‐dependent wound closure. Inflammation modestly reduced early migration, consistent with impaired motility observed in atopic keratinocytes [63]. All treatments enhanced migration at intermediate time points, indicating a general capacity to support re‐epithelialization under inflammatory stress. However, only hemp oil sustained a significant pro‐migratory effect at 24 h, resulting in markedly accelerated wound closure compared with both inflamed and control cells.

This sustained migratory response aligns with the pronounced barrier‐restorative effects of hemp oil and suggests coordinated regulation of junctional plasticity and cell motility [64, 65]. The more transient effects observed with BCS oil and vitamin D_3_ are consistent with previous evidence supporting their involvement in early phases of wound repair [66, 67, 68, 69, 70, 71] but suggest limited capacity to counteract prolonged inflammatory inhibition of migration. The accelerated wound closure observed in treated cells is unlikely to reflect the cytokine‐induced cellular hypertrophy discussed earlier, since wound closure was quantified as percent area covered relative to the initial scratch within each individual well, a metric that is intrinsically independent of individual cell size and reflects the net advancement of the wound edge over time.

Oxidative and nitrosative stress play a recognized pathogenic role in atopic dermatitis, where increased reactive oxygen and nitrogen species coexist with a functionally insufficient endogenous antioxidant response, contributing to barrier dysfunction and to the chronic engagement of inflammatory signaling [17, 45, 72]. In keeping with this background, the cytokine cocktail produced in our model a marked accumulation of 3‐nitrotyrosine in inflamed cells, confirming a sustained nitrosative protein damage at the 48‐h timepoint and providing a functional readout of the redox imbalance generated by the inflammatory stimulus. The choice to focus subsequently on the NRF2 transcriptional axis was based on the central role of this pathway in coordinating the cytoprotective antioxidant response in keratinocytes and on direct evidence that its activity is reduced in the epidermis of AD patients [20, 21]. Additional rationale was provided by previous reports describing the modulation of this axis by natural compounds with cytoprotective activity in keratinocytes, including cannabidiol [31]. The four treatments produced a clearly differential pattern of modulation along the NRF2/BACH1/HMOX1 axis. Hemp oil acted primarily on the upstream/activator arm, with a significant induction of *NFE2L2* mRNA above control levels and maintained NRF2 protein, whereas the downstream output of the pathway, *HMOX1* mRNA was not further increased and the transcriptional repressor *BACH1* was not modulated. The data therefore document, in our acute monolayer setting, a partial engagement of the cannabidiol–NRF2 link previously described by Casares and colleagues in primary human keratinocytes [31, 51]: NRF2 activation was present, but BACH1 degradation and HMOX1 induction were not observed. This divergence likely reflects the different experimental matrix (canine immortalized CPEK monolayer vs. primary human keratinocytes), the acute cytokine challenge, and the 48‐h timepoint, at which the early peak of *HMOX1* induction described in vitro may already have declined. Vitamin D_3_ and the combined formulation, in contrast, acted preferentially on the downstream arm of the axis, with significant induction of *HMOX1* and a marked reduction of *BACH1* approaching the conventional significance threshold, in line with the recognized role of VDR signaling in relieving transcriptional repression and enhancing the antioxidant output of keratinocytes. BCS oil did not significantly modulate any component of the axis. Two observations deserve specific framing: the persistence of 3‐nitrotyrosine and the maintenance or, for hemp oil, the further increase of NRF2 protein in treated cells. Neither finding should be read as evidence that the treatments worsen oxidative damage. 3‐NT is a cumulative footprint of protein nitration generated during the initial 24 h of cytokine exposure, with a protein‐turnover‐limited persistence longer than the 24 h treatment window; the dataset informs on the reversal of established nitration at the late timepoint, not on whether the treatments limit ongoing nitration. The sustained NRF2 protein elevation, similarly, is a canonical response to oxidative challenge and reflects an antioxidant program actively engaged in response to the inflammatory stimulus rather than compound‐induced toxicity. For hemp oil specifically, the parallel induction of *NFE2L2* mRNA above control values indicates a direct activator role on the NRF2 axis rather than a stress‐driven compensatory response.

The ELISA‐based quantification of secreted mediators provides functional evidence that the cytokine cocktail engages the major signaling axes operative in CPEK cells and reveals, at the same time, that the model recapitulates a clinically relevant inflammatory output. The robust induction of CXCL9 (three‐fold) and CXCL10 (six‐fold) in inflamed cells reflects the engagement of the IFN‐γ/STAT1 axis and is consistent with the well‐documented role of these CXCR3‐binding chemokines in recruiting Th1 and cytotoxic CD8^+^ T cells to the lesional skin, particularly in the chronic phase of AD and in intrinsic AD endotypes [15, 73]. In keratinocytes specifically, IL‐4 has been shown to potentiate the action of IFN‐γ in inducing these CXCR3 ligands, providing a mechanistic link between the Th2‐skewed initiation phase and the Th1‐amplified chronicisation of the disease, a process that our acute model captures by combining IFN‐γ, IL‐4, and IL‐13 within the same cocktail. The NF‐κB‐dependent branch, captured by IL‐8 (CXCL8) and IL‐6 secretion, was also significantly engaged, although with a smaller amplitude: IL‐8 is a recognized mediator of neutrophil recruitment in inflammatory skin diseases and is among the chemokines that contribute to the innate inflammatory component of AD, while IL‐6 produced autocrinously by keratinocytes sustains epidermal hyperplasia and acts as a downstream amplifier of STAT3 signaling, contributing both to disease chronicisation and to the keratinocyte hypertrophy discussed in previous sections [15]. CCL17/TARC, included as a STAT6‐specific readout, was below the lower limit of quantification of the assay in all conditions; the engagement of STAT6 by the IL‐4/IL‐13 components of the cocktail is, however, indirectly supported by the marked downregulation of CLDN1, a gene consistently suppressed by the IL‐4/IL‐13–JAK‐STAT6 axis in keratinocytes [74].

The pattern of treatment‐induced modulation maps onto a clearly pathway‐specific footprint, which translates directly into distinct potential immunological consequences. Hemp oil reduced CXCL9 and CXCL10 secretion but did not significantly modify IL‐8 or IL‐6, indicating a preferential action on the STAT1 chemokine arm and, by extension, a potential to limit the IFN‐γ/CXCR3‐driven recruitment of Th1 and CD8^+^ cells without significant suppression of the NF‐κB‐dependent innate inflammatory branch. BCS oil produced a mirror‐image profile, significantly reducing IL‐8 without normalizing the STAT1‐driven CXCL10, consistent with a γ‐linolenic acid‐mediated suppression of NF‐κB‐dependent pathways and with a potential preferential effect on the innate, neutrophil‐recruiting component of the response. Vitamin D_3_ and the combined formulation acted broadly across both axes, reducing CXCL9, CXCL10, and IL‐8, in line with the well‐documented broad‐spectrum anti‐inflammatory action of VDR signaling on multiple keratinocyte pathways and with the potential to dampen both the adaptive Th1 and the innate components of the inflammatory output. The convergence of these results with the proliferation, differentiation, barrier, and redox readouts discussed above reinforces the picture of four natural formulations modulating distinct but partially complementary nodes of the inflamed keratinocyte phenotype.

## 5 Limitations and Future Directions

Several limitations should be acknowledged when interpreting these findings. First, this study relied on an immortalized keratinocyte line and an in vitro cytokine‐based inflammatory model, which cannot fully recapitulate the cellular heterogeneity, immune–epithelial interactions, and chronic environmental influences present in canine atopic skin in vivo. While CPEK cells reproduce many hallmark features of cAD [75, 76, 77, 78], disease‐specific behaviors of primary keratinocytes [79] and contributions from immune cells, fibroblasts, and the microbiome remain unaddressed. Second, although the combined oil and vitamin D_3_ formulation demonstrated beneficial effects, the study was not designed to formally distinguish additive from synergistic interactions or to optimize formulation ratios. Third, the extension of the redox characterization to BACH1 quantification and to additional NRF2 transcriptional targets (NQO1, GCLC, GCLM) would strengthen the mechanistic interpretation of the differential modulation of the NRF2/BACH1/HMOX1 axis observed here.

Future studies should therefore extend these findings to primary keratinocytes derived from atopic dogs, incorporate co‐culture systems with immune cells, and validate barrier and wound‐healing effects in ex vivo or in vivo models. Detailed mechanistic analyses dissecting the contributions of specific hemp oil constituents, including cannabidiol and lipid components, will be essential to refine formulation strategies. Ultimately, controlled clinical studies will be required to determine whether the barrier‐supportive and antioxidant effects observed here translate into meaningful therapeutic benefits for dogs with atopic dermatitis.

## Funding

This work was supported by NBF Lanes.

## Supporting information


**Figure S1:** Flow‐cytometry gating strategy for Ki‐67, NRF2 and cell‐cycle analyses. (A) Sequential gating strategy applied to all flow‐cytometry experiments. Cells were first identified by their forward‐scatter (FSC‐A) and side‐scatter (SSC‐A) properties to exclude debris, then single cells were selected by the FSC‐H/FSC‐A plot to exclude doublets, and finally viable cells were gated as live/dead‐marker‐negative events (LD APC‐A750‐A vs. SSC‐A). Representative dot‐plots are shown together with the percentage of events retained at each gating step.(B) Representative fluorescence histograms showing the gating used to quantify Ki‐67 (left, Alexa Fluor 647) and NRF2 (right, PE). Sample distributions (red) are shown overlaid on the corresponding fluorescence‐minus‐one (FMO) controls (cyan), used to define the positive populations. Ki‐67‐positive cells were quantified as the percentage of events above the FMO threshold, and NRF2 protein levels were quantified as median fluorescence intensity (MFI) of the NRF2 channel within the live, single‐cell population.(C) Representative DNA‐content histogram for cell‐cycle analysis. Propidium iodide (PI) fluorescence intensity (FL8‐A) was used to identify the G_0_/G_1_, S, and G_2_/M cell‐cycle phases, with the corresponding subpopulations highlighted by the colored peaks (Watson cell‐cycle model fit). The gates used to define the G_0_/G_1_ and G_2_/M populations are indicated by the gray vertical bars.
**Figure S2:** Effects of hemp oil, blackcurrant seed oil, vitamin D_3_, and their combination on keratinocyte proliferation assessed by MTT assay. CPEK cells were treated with increasing concentrations of the indicated compounds and cell proliferation was evaluated by MTT assay. (A) Hemp oil, expressed as percentage of cell proliferation relative to untreated controls, plotted against the logarithm of oil dilutions. (B) Blackcurrant seed oil, expressed as percentage of cell proliferation relative to controls, plotted against the logarithm of oil dilutions. (C) Vitamin D_3_, expressed as percentage of cell proliferation relative to controls, plotted as a function of increasing concentrations. (D) Combination of hemp oil and blackcurrant seed oil with vitamin D_3_, expressed as percentage of cell proliferation relative to controls, plotted against the logarithm of oil dilutions. Data are reported as mean ± SEM of 3 independent experiments.
**Figure S3:** Effects of hemp oil, blackcurrant seed oil, vitamin D_3_, and their combination on keratinocyte viability following cytokine‐induced inflammation. CPEK cells were exposed for 24 h to a cytokine cocktail representative of the inflammatory milieu associated with canine atopic dermatitis and subsequently treated with hemp oil, blackcurrant seed oil, vitamin D_3_, or their combination. Cell viability was assessed by Trypan Blue exclusion assay and expressed as percentage of viable cells relative to cytokine‐treated controls. Data are reported as mean ± SEM of 3 independent experiments.

## Data Availability

The data that support the findings of this study are available from the corresponding author upon reasonable request.
