# Supplementary material for: Differential Modulation of Keratinocyte Proliferation, Differentiation, and Barrier Function by Hemp Oil, Blackcurrant Seed Oil, and Vitamin D3 Under Atopic‐Associated Cytokine Stimulation
Source: Biofactors. 2026 Aug 10;52(4):e70137. doi: 10.1002/biof.70137 (PMC13457580; doi:10.1002/biof.70137)
Supplement: Supplementary file 1 [file BIOF-52-0-s001.docx]

**Differential Modulation of Keratinocyte Proliferation, Differentiation, and Barrier Function by Hemp Oil, Blackcurrant Seed Oil, and Vitamin D₃ under Atopic-Associated Cytokine Stimulation**

Tortolani D.^1,2#^, Scipioni L.^1,2^, Anselmucci A.^1^, Ciaramellano F.^1,2^,

Di Leonardo M.^3^, Fusaro I. ^1^, Oddi S.^1,2#^, Gramenzi A.^1^

1. Department of Veterinary Medicine, University of Teramo, 64100 Teramo, Italy.

2. European Center of Brain Research/Fondazione Santa Lucia IRCCS, 00143 Rome, Italy,

3. Azienda Sanitaria Locale di Teramo, 64100 Teramo, Italy.

#corresponding author

**Supplementary material**

**Figure S1. Flow-cytometry gating strategy for Ki-67, NRF2 and cell-cycle analyses.** (**A**) Sequential gating strategy applied to all flow-cytometry experiments. Cells were first identified by their forward-scatter (FSC-A) and side-scatter (SSC-A) properties to exclude debris, then single cells were selected by the FSC-H/FSC-A plot to exclude doublets, and finally viable cells were gated as live/dead-marker-negative events (LD APC-A750-A vs SSC-A). Representative dot-plots are shown together with the percentage of events retained at each gating step.(**B**) Representative fluorescence histograms showing the gating used to quantify Ki-67 (left, Alexa Fluor 647) and NRF2 (right, PE). Sample distributions (red) are shown overlaid on the corresponding fluorescence-minus-one (FMO) controls (cyan), used to define the positive populations. Ki-67-positive cells were quantified as the percentage of events above the FMO threshold, and NRF2 protein levels were quantified as median fluorescence intensity (MFI) of the NRF2 channel within the live, single-cell population.(**C)** Representative DNA-content histogram for cell-cycle analysis. Propidium iodide (PI) fluorescence intensity (FL8-A) was used to identify the G₀/G₁, S, and G₂/M cell-cycle phases, with the corresponding subpopulations highlighted by the coloured peaks (Watson cell-cycle model fit). The gates used to define the G₀/G₁ and G₂/M populations are indicated by the grey vertical bars.

**Figure S2. Effects of hemp oil, blackcurrant seed oil, vitamin D₃, and their combination on keratinocyte proliferation assessed by MTT assay.** CPEK cells were treated with increasing concentrations of the indicated compounds and cell proliferation was evaluated by MTT assay. (A) Hemp oil, expressed as percentage of cell proliferation relative to untreated controls, plotted against the logarithm of oil dilutions. (B) Blackcurrant seed oil, expressed as percentage of cell proliferation relative to controls, plotted against the logarithm of oil dilutions. (C) Vitamin D₃, expressed as percentage of cell proliferation relative to controls, plotted as a function of increasing concentrations. (D) Combination of hemp oil and blackcurrant seed oil with vitamin D₃, expressed as percentage of cell proliferation relative to controls, plotted against the logarithm of oil dilutions. Data are reported as mean ± SEM of 3 independent experiments.

**Figure S3. Effects of hemp oil, blackcurrant seed oil, vitamin D₃, and their combination on keratinocyte viability following cytokine-induced inflammation.** CPEK cells were exposed for 24 h to a cytokine cocktail representative of the inflammatory milieu associated with canine atopic dermatitis and subsequently treated with hemp oil, blackcurrant seed oil, vitamin D₃, or their combination. Cell viability was assessed by Trypan Blue exclusion assay and expressed as percentage of viable cells relative to cytokine-treated controls. Data are reported as mean ± SEM of 3 independent experiments.
